# Disposable cartridge concept for the on-demand synthesis of turbo Grignards, Knochel–Hauser amides, and magnesium alkoxides

**DOI:** 10.3762/bjoc.16.115

**Published:** 2020-06-19

**Authors:** Mateo Berton, Kevin Sheehan, Andrea Adamo, D Tyler McQuade

**Affiliations:** 1 Department of Chemical and Life Sciences Engineering, Virginia Commonwealth University, Biotech Eight, 737 N. 5th St., Box 980100, Richmond, VA 23219, USA; 2 Zaiput Flow Technologies, 300 2nd Avenue, Waltham, MA 02451, USA

**Keywords:** Knochel–Hauser base, lithium chloride, magnesium, on-demand, packed-bed reactors, plug and flow reactor, synthesizer, turbo Grignard reagent

## Abstract

Magnesium organometallic reagents occupy a central position in organic synthesis. The freshness of these compounds is the key for achieving a high conversion and reproducible results. Common methods for the synthesis of Grignard reagents from metallic magnesium present safety issues and exhibit a batch-to-batch variability. Tubular reactors of solid reagents combined with solution-phase reagents enable the continuous-flow preparation of organomagnesium reagents. The use of stratified packed-bed columns of magnesium metal and lithium chloride for the synthesis of highly concentrated turbo Grignards is reported. A low-cost pod-style synthesizer prototype, which incorporates single-use prepacked perfluorinated cartridges and bags of reagents for the automated on-demand lab-scale synthesis of carbon, nitrogen, and oxygen turbo magnesium bases is presented. This concept will provide access to fresh organomagnesium reagents on a discovery scale and will do so independent from the operator’s experience in flow and/or organometallic chemistry.

## Introduction

Flow chemistry has facilitated: (1) new applications of high-energy or otherwise unsafe chemistry [1–2], enabled by a controlled/rapid heat removal and generation and the immediate use of unstable species [3–4]; (2) flash chemistry, where rapid mixing can outcompete unimolecular side reactions [5–6]; (3) new chemistry by conducting reactions outside of normal operating pressures and temperatures [7–8]; (4) new opportunities for the realization of automated chemistry, including on-demand systems [9–12]. We have recently focused on systems where solid-reagent cartridges are combined with a solution-phase reagent, including: (1) copper(I) oxide to produce N-heterocyclic carbene–Cu(I) complexes for use as catalysts [13]; (2) proline to perform proline-based catalytic reactions [14]; (3) zinc powder to produce organozinc halides in tandem with Negishi couplings [15]; (4) zinc complexes to produce fluorescent species [16]; (5) sodium borohydride to reduce carbonyls [17]; (6) red phosphorous to produce polyphosphides [18]. Our initial foray into this area was born out of necessity. We wanted to conduct flow reactions that required solids, and packed beds facilitated the use of solids without clogging. More recently, we began to think about this combination for producing air- and water-sensitive reagents immediately prior to use. In particular, we were interested in addressing a dichotomy where discovery-scale (50–100 mL) organometallic reagents are used with uncertain characteristics, as opposed to a large scale where the specs are often defined for all reagents, including organometallics. The hypothesis is that unstable/unsafe reagents can be synthesized and used as needed for this discovery scale instead of purchasing stock solutions that arrive with uncertain properties and require titration to determine the concentrations.

**Figure 1:**
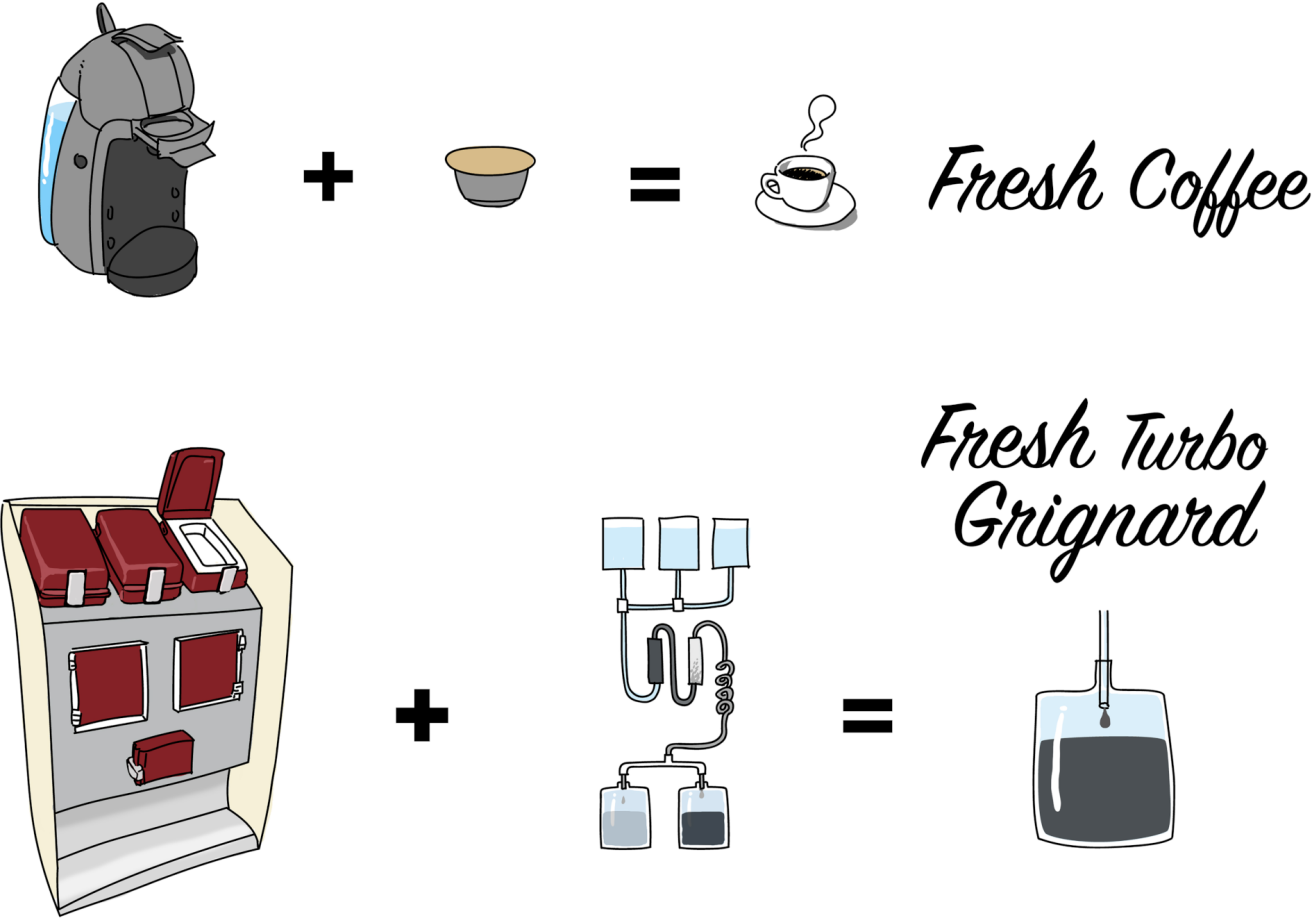
Comparing on-demand coffee and turbo Grignard pod-style machines.

Both commercial and academic flow systems are commonly constructed for experienced flow chemists and are designed to maximize a versatile operation to explore a broad range of chemical transformations [11,19–30]. These systems are designed to achieve a generality of operation, and this comes with an increase in the cost and complexity of the instruments. Our on-demand approach targets the opposite end of the equipment design spectrum; it requires a low-cost systems designed to carry out only a few specific functions in a safe and robust manner. It also demands to be low-cost in order to have any potential for real-world applications. In other words, to achieve the set goals, innovation is needed to reduce the complexity/expense of (1) pumps; (2) reactors; (3) valves; (4) fittings, and (5) chemical containers. The design presented here is based on a disposable cartridge concept, inspired by pod-based coffee machines (Figure 1). We took inspiration from recent efforts that demonstrated that simple machines can do valuable chemistry [12]. Our “cartridge” encompasses reagent bags, tubing, and packed-bed columns of solid reagents and product receptacle. These components are deployed in a low-cost machine with a design amenable for the automated lab-scale generation of organomagnesium reagents on demand (Figure 1).

Organomagnesium compounds are omnipresent reagents that serve as nucleophiles and bases. Grignard reagents react with oxygen and water, yielding flammable gases and must be prepared, stored, and handled under an anhydrous inert atmosphere. Time-consuming titration is recommended but is unreliable as only the basicity is estimated. The freshness of these solutions is a key for achieving high conversion because the neutralization can alter the aggregation states, producing a significant batch-to-batch variability. The direct insertion of magnesium metal into organic halides is the most common method used to prepare Grignard reagents but present difficulties: (1) sluggish reactions with ordinary magnesium turnings [31]; (2) the formation of undesired side products by thermal decomposition and exothermic reactions not suitable for industrial processes [32]; (3) the activation of a metallic surface is required and can introduce safety issues due to the high reactivity of the activated metal.

Flow chemistry technologies and cartridges containing activated metals can solve most of these issues: (1) the use of activated magnesium powder packed in a column increases the reaction rate and facilitates safe separation of the metal and reagent solution; (2) an efficient heat transfer (a large surface area-to-volume ratio) provides thermal control during metal activation and the generation of concentrated organometallic solutions; (3) the control over the residence time reduces side products because the organometallic solution is not exposed to high temperatures longer than necessary. All these advantages allow a more straightforward production and use of these critical reagents.

The preparation of organozinc species using zinc packed-bed columns [15,33–35] provides examples for the progression toward the on-demand synthesis of other organometallic reagents. While the concept of a reactive packed bed is not new, many features must be considered and solved for success, including: (1) the column packing–making sure the particle size range and how the column is packed provides a system with minimal channeling; (2) selecting a column with the right properties, such as the materials of construction, pressure tolerance, heat conduction, and diameter/particle size matching; (3) the column orientation and setup–filters, etc.; (4) activation of the solid phase. The activation issue is one of the most important factors when considering the metal packing. Although our team had success with zinc packing, we still need to develop a new approach for magnesium. Magnesium, when activated, is more reactive compared to zinc, in part because magnesium is a stronger reducing agent than zinc. Beyond the considerations of the packing, column, and activation, the solubility of organomagnesium reagents is often lower than of the corresponding zincates. The low solubility can clog the column or may reduce the insertion rate by forming a passivating layer over the metal particle surface.

Few examples describe the production of organomagnesium species under flow conditions [36–39], and only three use a practical system with a broad range of substrates [40–42]. The Alcázar group reported the generation and subsequent use of Grignard reagents [40]. In 2018, the Loren group extended the scope of the organozinc reagents made in flow to aryl and tertiary alkyl halides by the in situ formation of the corresponding Grignard intermediate in the presence of ZnCl_2_ and LiCl, which were subsequently used in Negishi cross-coupling reactions [41]. The most recent example, by the Löb group, reported a pilot plant reactor including a Mg replenish unit that allowed to generate phenylmagnesium bromide (1 M) at flow rates up to 15 L/h [42].

**Figure 2:**
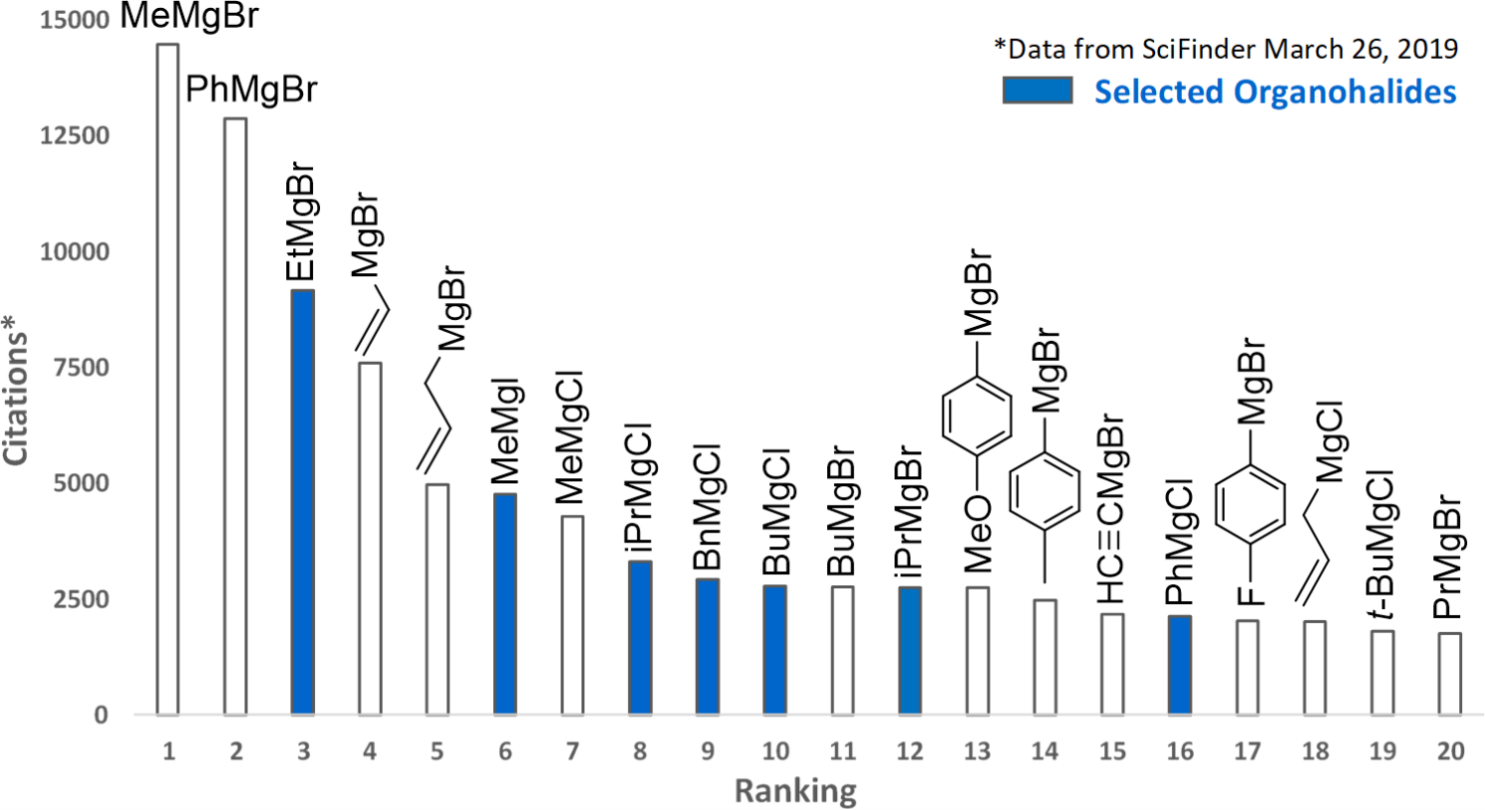
Ranking of the 20 most cited Grignard reagents (SciFinder March 26, 2019).

However, in these publications, alkyl chloride substrates, which are generally more cost-effective but less reactive than the corresponding bromide or iodide, are limited. Also, the use of a LiCl solution as the reaction medium to increase the Grignard reagent solubility was prepared from hygroscopic LiCl, which implies thorough drying and storage under a moisture-free atmosphere. Finally, the concentration of the Grignard reagents was limited, being in the range of 0.3–0.5 M, thereby limiting the range of reaction conditions for the discovery chemist. In this study, we started selecting some of the most used organomagnesium halides in synthesis. For this purpose, a ranking of the 20 most cited ones, as measured by the citation values obtained from SciFinder, was constructed (Figure 2). Based on our analysis, methyl, ethyl, isopropyl, butyl, benzyl, and phenyl as the residue R were selected as test cases for our proposed system.

Over the last two decades, Knochel demonstrated the benefits of LiCl on the halogen–magnesium exchange rates [43] and on the organomagnesium solubility [44]. The most known example of this class is the isopropylmagnesium chloride–lithium chloride complex (iPrMgCl⋅LiCl), known as turbo Grignard [45]. In addition to being widely cited, turbo Grignard is a popular discovery-scale tool in the pharmaceutical industry [32] and has shown an excellent selectivity on a large scale [46]. Halomagnesium amide LiCl adducts, e.g., the Knochel–Hauser base (TMPMgCl⋅LiCl), are also useful reagents for selective deprotonations due to their strong basicity and low nucleophilicity [47–48]. Knochel-type alkoxides [49], e.g., the 2-methyl-2-propoxymagnesium chloride–lithium chloride complex (*tert*-AmylOMgCl⋅LiCl), are less common in synthesis, but their high reactivity and solubility, combined with a high tolerance towards functional groups, made them advantageous for selective transformations.

Herein, a novel disposable cartridge approach for the on-demand, discovery-scale preparation of turbo Grignard reagents, Knochel–Hauser bases, and new Knochel-type alkoxides using a stratified bicomponent packed-bed column of magnesium and lithium chloride is presented. Critical insights, such as column packing, particle size, metal excess, reagent scope, order of addition, column stability, reproducibility, and consideration of solid/liquid reaction models are presented. In addition, a proof-of-concept, automated pod-type synthesizer prototype designed to generate up to 100 mmol of fresh reagents on demand is described. Our objective is to help others integrate this approach into their quotidian workflow to enable discovery-scale researchers to increase the reliability of their developed routes and processes by increasing the quality of their organomagnesium reagents.

## Results and Discussion

### 1 On-demand reagent prototype

**Objective:** To design and create a simple, robust, disposable, and low-cost system capable of producing on-demand reagents for lab-scale purposes using a combination of liquid and solid pods or cartridges. We envisioned a system that requires: (1) pumps; (2) tubular reactors; (3) valves; (4) fittings, and (5) chemical containers.

**Challenges:** The need to develop new design concepts to achieve (1) low-cost high-pressure pumps (10 bar); (2) disposable tubular reactors; (3) robust valves; (4) leakproof bonding process, and (5) chemically compatible and high-pressure containers.

**Figure 3:**
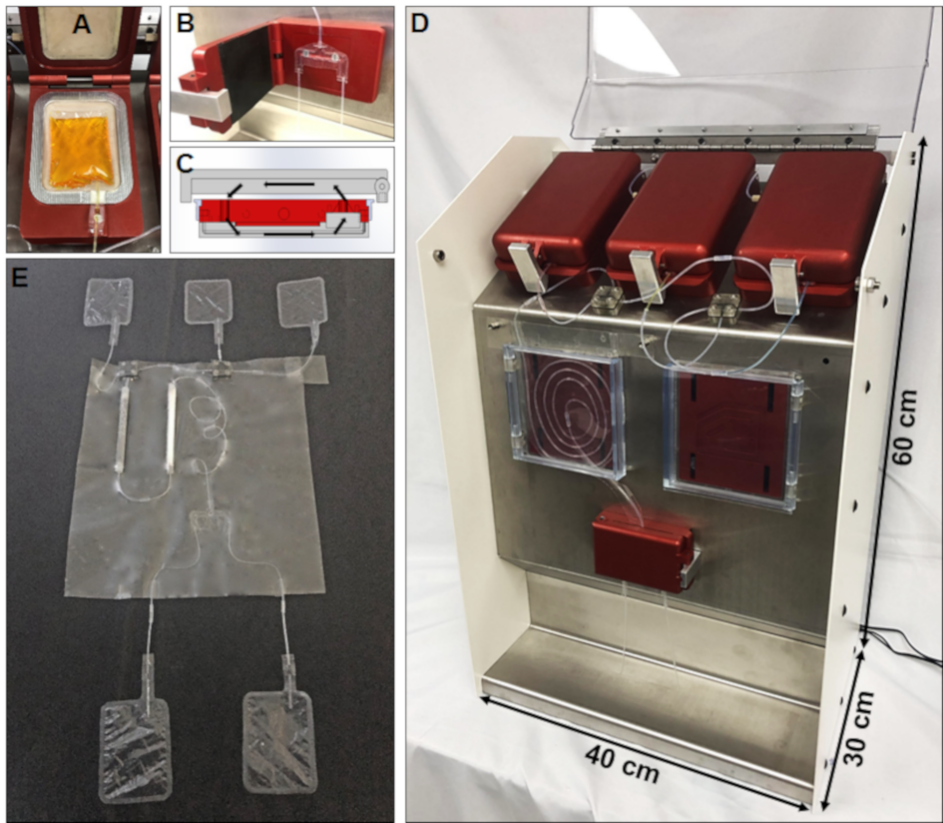
On-demand prototype. A) Inside view of the pump with a flexible bag containing a yellow liquid laying on an elastomer membrane. B) Detail of the manifold used to select the waste or product collection. C) Heater cross section, the arrows indicate the air convection flow path. D) Reagents on-demand system assembled, with the coil reactor placed in the left reactor area. E) Example of a disposable cartridge.

**System design:** The pod-style concept is achieved by making the entire fluidic circuit that is needed to run a specific chemistry with prearranged, custom-made, and thermally bonded parts. Parts were built of fully perfluorinated materials, providing an excellent chemical resistance. We chose thermal bonding because this type of bond can provide a leakproof system without the need of fittings. All the fluidic items and reagents assembled together represent the “cartridge” (Figure 3E). Prebuilt disposal cartridges have a long shelf life and can be deployed on demand. The instrument we produced provides the necessary pumping (Figure 3A), heating (Figure 3C), and valving (Figure 3B), which are united in an enclosed unit that can be loaded with self-contained cartridges (Figure 3E). In order to build the disposable cartridges, we developed bonding protocols to carry out the different types of connections needed (i.e., tube to tube, tube to cartridge, tube to bag, tee, etc.).

**Pump concept:** The pumping is achieved by developing flexible reservoirs made with perfluorinated polymer film (PFA), and the bags are filled with the fluid to dispense. The pumping is accomplished by enclosing the bag in a metal clamshell, with contact surfaces made with an elastomer (Figure 3A). When compressed air is pumped in the clamshell, it squeezes the bag through the elastomer, and thus dispensing fluid. The elastomer sheets fully embrace the reagent bag, providing mechanical support. In this way, a soft polymer bag can be squeezed at a relatively high pressure: our prototype achieved pumping pressures up to about 1 MPa (10 bar). This type of pressure-driven pump, where the dispensed liquid is enclosed in a plastic reservoir, provides the advantage of not dissolving any gas into the liquid during the operation, which is the case for pressurized tanks. Additionally, all the wetted parts of the pump are fully disposable (Figure 3E). The metering function required to keep the back pressure and a constant flow rate is achieved by the tubing length and diameter downstream of the pump or by the fluidic network for more complex cases. The flow is pulseless as a result of the fact that it is driven by compressed air.

**On-demand reagent (ODR) system design:** The ODR prototype (Figure 3D) is essentially composed of three clamshell pumps in order to have up to three process fluids. Each bag contains a valving, so that only specified fluids can be dispensed when required by the process. The instrument offers up to two reaction areas made by aluminum plates. The convection of air is generated with small fans embedded in the aluminum plate to improve the quality of the heat transfer to the reactor. A manifold (Figure 3B) is placed downstream of the reaction areas where actuators control the direction of the outcome stream. The solvent priming and the activation solution are discarded into the waste, and only when product is generated, the manifold starts to collect. The system’s heating, temperature control, and valving are controlled by an Arduino card (not shown–on the back of the system). Different control routines can be loaded into the Arduino card as needed.

### 2 Grignard reagents via magnesium-packed beds

**Objective:** To generate concentrated organomagnesium solutions from alkyl halides using a standardized and reproducible packed bed of magnesium, to develop a consistent activation protocol using a single activation solution, and to optimize the conditions for the quantitative organic chloride conversion.

**Challenges:** Metal surface activation, organomagnesium solubility, formation of a black side product, performance, and degradation over time.

**System setup:** A commercial flow chemistry system [50] equipped with a temperature-controlled glass manifold reactor [51] was used (Figure S1, Supporting Information File 1). We have found that both glass and PFA columns with similar dimensions can be used. To reduce the costs, the flow chemistry system can be replaced by syringe or HPLC pumps, and the reactor heating can be accomplished using standard heating tools (water/oil bath, heating jacket, or a suitable oven). The 10 × 100 mm (ID × length) column was filled with magnesium. A back-pressure regulator (BPR) was added to prevent the gas/liquid separation and to increase the solvent boiling temperature.

We started reproducing Alcázar’s conditions to obtain organomagnesium bromide reagents from the corresponding alkylbromides. The activation protocol was slightly modified: a single activating solution composed of 1-bromo-2-chloroethane, TMSCl, and DIBAL-H in THF/toluene was pumped through magnesium powder (98%, 20–230 mesh) at 1 mL/min and 40 °C (Section 1.2.2 in Supporting Information File 1). Organomagnesium bromide reagents (15 mL) were generated in THF at a 0.5 mL/min flow rate and 25 °C. For each experiment, the concentration was determined in duplicates by the reaction with a known mass of two different indicators until a color change occurred: 2-hydroxybenzaldehyde phenylhydrazone [52] or a mixture of benzoic acid and 4-(phenylazo)diphenylamine [53]. Iodine can also be used [54]. Similar concentrations were obtained by NMR titration with 1,5-cyclooctadiene as a standard (Section 1.2.10 in Supporting Information File 1) [55].

**Figure 4:**
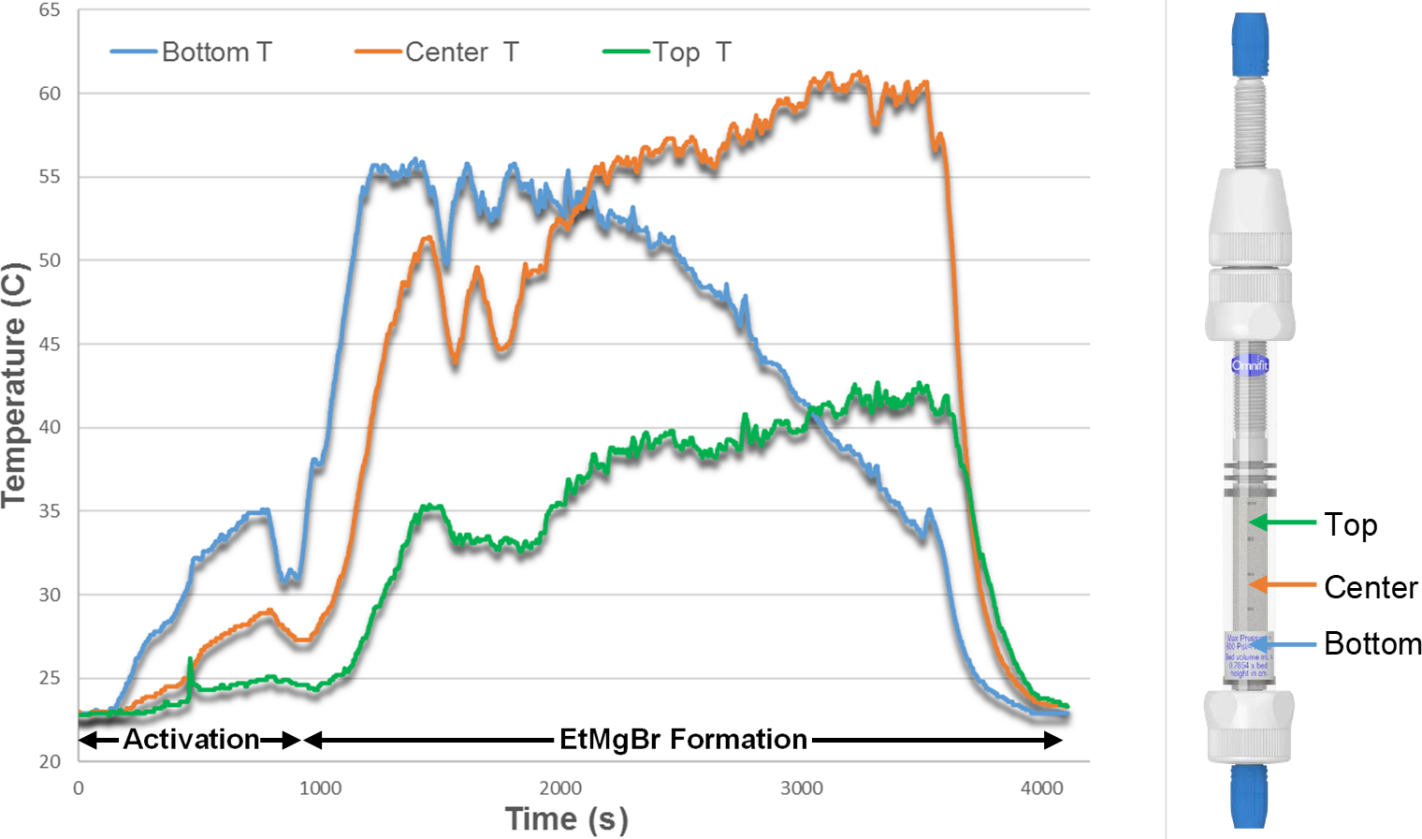
Temperature evolution measured with thermocouples along the column outer surface at three different points.

The heat released from the exothermic Grignard reaction done at 25 ºC was not fully dissipated by the heat exchanger (≈10 ºC increment). To better understand this exothermic process, we decided to measure the temperature evolution during the conversion of EtBr (0.5 M) at a 0.5 mL/min flow rate with no heat controller. Three thermocouples were placed at different points along the glass column (Figure 4 right). The results showed a ≈35 °C increment, which is an underestimation because the measurements were taken on the outer surface of the glass column. The data featured a hot spot moving upward during the reaction (Figure 4 left), suggesting that magnesium was consumed by the layers at a 0.5 mL/min flow rate, and the reaction occurred almost exclusively at the interphase EtBr–Mg* and not along the whole column. Since the reactive interphase moved upward at the same rate that Mg was getting consumed, the heat release was not constant along the column, and a steady-state temperature only occurred during a short amount of time at a localized area. The temperature gradient was not observed at higher temperatures (60–100 ºC), and the hot spot generated was less than +5 ºC under these conditions.

During the isopropylmagnesium bromide optimization (Table S4, Supporting Information File 1), solubility issues hindered the formation of a concentrated solution (>0.8 M). The crystallization of iPrMgBr in the collection flask forced us to reduce the initial concentration of the organic halide to 0.9 M, yielding iPrMgBr (0.75 M, 82%, note: the yields reported herein refer to the amount of organometallic reagent produced in a steady state; no detectable halide was recovered, and the organometallic reagent purity was high unless otherwise stated, Table S4, entry 3, Supporting Information File 1). The reactivity order of organic halides against oxidative addition reactions is R–I > R–Br > R–Cl. To achieve the direct insertion of 2-chloropropane, the temperature was increased, and the best result was obtained at 80 °C, yielding iPrMgCl (0.78 M, 87%, Table S4, entry 7, Supporting Information File 1). Since iPrMgCl is more soluble in THF than the corresponding bromide [56], we were able to use an initial concentration of up to 2.5 M, yielding iPrMgCl (2.23 M, 89%, Table S4, entry 8, Supporting Information File 1). At a temperature higher than 60 °C, 100 psi BPR were required to prevent the solvent from boiling inside the packed-bed column.

During preliminary experiments, we observed the formation of a black residue that was left after the magnesium consumption. While the residue did not affect the column performance for the conversion of 10 mL of an iPrCl (2.5 M) solution, larger generated volumes increased the pressure drop and eventually led to clogging of the system. The analysis of the black residue via X-ray photoelectron spectroscopy (Figure S6, Supporting Information File 1) revealed the presence of magnesium, oxygen, carbon, and chlorine. Although we do not understand the mechanism, we sought a solution that would enable the column performance to remain constant. During the optimization, we tested Mg chips (99.98%, 6–35 mesh) and observed less black residue. Thus, we explored the reaction using different ratios of Mg chips/powder (Figure S7, Supporting Information File 1). We found that a Mg chip/powder ratio of 1:1 provided more consistent results over a relatively large amount (≈100 mmol) of organic halide that was converted. We offer two explanations: (1) the higher purity of Mg chips (99.98%) and (2) the higher surface area (SA) of the Mg powder (≈130 mesh, SA ≈ 30 cm^2^/g) than Mg chips (≈20 mesh, SA ≈ 4 cm^2^/g). We hypothesize that these features provided a large activated Mg surface for an initial quantitative conversion and a purer but less reactive material that generated less side products, resulting in an iPrMgCl yield of up to 97%. The optimal amount of Mg was determined to be 2 equivalents. The yield drop after the consumption of 1 equiv of Mg was first attributed to a channeling through the packed bed. To test this hypothesis, we followed the changes in the column using a 4K webcam for two types of columns: (1) a firmly packed column and (2) a loosely packed column. The well-packed Mg column did not consume all the Mg due to the channeling (Figure S8A, Supporting Information File 1), as we proposed. The loosely packed column, to our surprise, behaved like a fluidized bed, allowing iPrCl to be in contact with a larger surface of Mg (Figure S8B, Supporting Information File 1) and provide a better performance than a well-packed column: 98%.

**System scope:** Next, we probed the limit of this transformation for primary (bromoethane, bromooctane, chlorobutane, and iodomethane) and secondary (2-bromopropane, 2-chloropropane, and 2-chlorobutane) alkyl halides as well as benzyl (chloromethylbenzene) and aryl chlorides (chlorobenzene).

Good to excellent yields were obtained (Table 1). In general, bromo-Grignard reagents tend to be less soluble in THF than chloro-Grignard reagents, and other ethereal solvents can be more appropriate: 2-methyltetrahydrofuran (2-MeTHF) or Et_2_O. The higher solubility of EtMgBr in these solvents allowed us to obtain 2.21 M (88%) in 2-MeTHF and 2.40 M (96%) in Et_2_O but only 1.08 M (90%) in THF (Table 1, entries 2–4). The concentration of n-OctMgBr was also limited to 0.51 M (85%) in THF (Table 1, entry 5). The use of Et_2_O increased the solubility up to 1.08 M (90%, Table 1, entry 6). Even chlorobenzene, considered a deactivated species, was converted to PhMgCl in an excellent yield (2.32 M, 93%, Table 1, entry 10), heating the column to 100 °C. For benzyl chloride, a 2-MeTHF/THF, 9:1 mixture [57] was found to be optimal to reduce the formation of a Wurtz-type side product, 1,2-diphenylethane (Table S5, Supporting Information File 1), yielding BnMgCl (0.99 M, 83%, Table 1, entry 11). Noteworthy, a iodomethane (bp 42 °C) solution in Et_2_O can be converted to the corresponding MeMgI in good yield and with a good mass balance using a 140 psi BPR (Table 1, entry 12).

**Table 1:** Reaction of organic halides in a packed-bed column of activated magnesium. Scope of Grignard reagents prepared under flow conditions.^a^

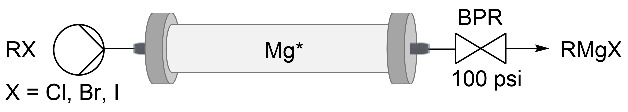						

entry	RX	*T* (*°*C)	solvent	[RX] (M)^b^	[RMgX] (M)	yield (%)^c^

1	iPrBr	25	THF	0.9	0.74	82^d^
2	EtBr	25	THF	1.2	1.08	90
3	EtBr	25	2-Me-THF	2.5	2.21	88
4	EtBr	25	Et_2_O	2.5	2.39	96
5	*n*-OctBr	25	THF	0.6	0.51	85
6	*n*-OctBr	25	Et_2_O	1.2	1.08	90
7	iPrCl	80	THF	2.5	2.38	95
8	*sec*-BuCl	80	THF	2.5	2.23	89
9	*n*-BuCl	80	THF	2.5	2.37	95
10	PhCl	100	THF	2.5	2.32	93
11	BnCl	60	2-Me-THF^e^	1.2	0.99	83^f^
12^g^	MeI	25	Et_2_O	2.5	2.36	94

^a^The RX solution (20 mL) was pumped at a 0.5 mL/min flow rate through a column (ID = 10 mm) of Mg* (2 equiv) chips/powder (1:1). ^b^Quantitative RX conversion. ^c^Determined by the titration of an overall RMgX solution (≈15 mL) collected at steady state. ^d^2-Methylpropene obtained as a major side product. ^e^10% of THF. ^f^1,2-Diphenylethane was obtained as a single side product. ^g^140 psi BPR.

### 3 Turbo Grignards via stratified packed-bed columns containing magnesium and LiCl

**Objective:** To generate organomagnesium–lithium chloride complexes (turbo Grignards) from alkyl chlorides using a stratified bicomponent packed-bed column composed of magnesium metal and lithium chloride.

**Challenges:** Metal passivation by lithium chloride coating, handling of LiCl (hygroscopic), LiCl equivalent optimization due to the solubilization over time.

**Figure 5:**
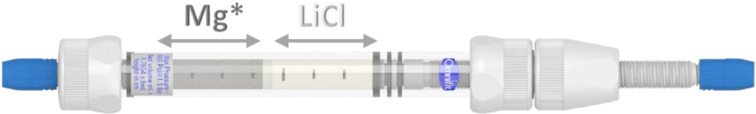
Stratified bicomponent column (Diba Omnifit EZ Solvent Plus) composed of magnesium (chips/powder, 1:1) and lithium chloride separated with fiberglass for the turbo Grignard reagent synthesis.

**System setup:** The same flow system was used as for the generation of Grignard reagents (Figure S2, Supporting Information File 1). The 10 × 100 mm (ID × length) column was half filled with magnesium (chips/powder, 1:1) and the second half with anhydrous lithium chloride (Figure 5). The two components were separated by fiberglass previously dried at 120 °C overnight. A 100 psi BPR was added to prevent the gas/liquid separation and to increase the solvent boiling temperature.

Clogging is a common concern in flow chemistry, and during our scope exploration, we observed that the concentrations of the organomagnesium reagents generated were mostly limited by their solubility. Knochel pioneered the use of lithium chloride to solubilize organometallic reagents and to increase the reactivity, most probably due to the disaggregation of oligomers [43–44]. We used this approach to overcome the solubility issue under continuous conditions. First, we verified that similar results are achieved in presence and absence of LiCl in solution for the EtMgBr formation (Section 2.2, Supporting Information File 1). Because organomagnesium halide–lithium chloride complexes are believed to be 1:1 RMgX⋅LiCl dimers, and considering the LiCl solubility limitation of ≈0.5 M in THF, we decided to design a new system for the generation of highly concentrated turbo Grignard reagents. Instead of using a solution of starting material and LiCl, a bicomponent packed-bed column was assembled. First, ≈4.5 cm of the Omnifit column was filled with magnesium (chips/powder, 1:1) and the upper ≈4.5 cm with anhydrous lithium chloride (Figure 5). The two components were separated by fiberglass previously dried at 120 °C overnight. We first tested this column with a 1.5 M EtBr solution, and a comparable EtMgBr⋅LiCl concentration was obtained (1.30 M, 87%, Table 2, entry 1) in comparison to dissolved LiCl (1.27 M, 86%, Table S6, entry 6, Supporting Information File 1).

**Table 2:** Reaction of organic halides with a stratified packed-bed column of activated magnesium and lithium chloride. Scope of turbo Grignard reagents prepared under flow conditions.^a^

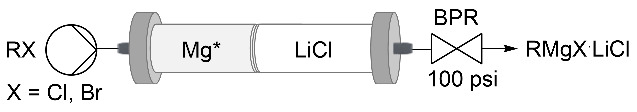					

entry	RX	*T* (°C)	[RX] (M)^b^	[RMgX⋅LiCl] (M)	yield (%)^c^

1	EtBr	25	1.5	1.30	87
2	iPrCl	80	2.5	2.19	88
3	*sec*-BuCl	80	2.5	2.15	86
4	*n*-BuCl	80	2.5	2.13	85

^a^The RX solution (15 mL) in THF was pumped at a 0.5 mL/min flow rate through a bicomponent column (ID = 10 mm) composed of activated Mg* (2 equiv) chips/powder (1:1) and anhydrous LiCl (2 equiv) separated by fiberglass. ^b^Quantitative RX conversion. ^c^Determined by the titration of the overall RMgX·LiCl solution (≈10 mL) collected at steady state.

The separation of the two components was crucial to obtain reproducible results. When Mg and LiCl were intimately mixed together, a reactivation of the column failed, likely due to magnesium surface passivation. The separation of Mg/LiCl allowed to reuse the column several times with different substrates. Nevertheless, we do not recommend its reutilization. For optimal results, 2 equivalents of Mg* (chips/powder, 1:1) and 2 equivalents of LiCl must be used at a single time.

**System scope:** The bicomponent column was employed to obtain the turbo Grignard reagent [45] as well as *sec*- and *n*-butylmagnesium chloride–lithium chloride complexes as THF solutions (≈10 mL). Very good yields were obtained: iPrMgCl⋅LiCl 2.19 M (88%); *s*-BuMgCl⋅LiCl 2.15 M (86%), and *n*-BuMgCl⋅LiCl (2.13 M, 85%, Table 2, entries 2–4).

The formation of the turbo Grignard reagent (iPrMgCl⋅LiCl) was scaled up to ≈100 mmol using a 15 × 100 mm column, and the results were compared with the Knochel batch protocol [58]. Using our flow procedure, the generation of a higher iPrMgCl⋅LiCl concentration (2.10 M instead of 0.89 M) in a shorter reaction time (1.5 h instead of 12 h) caused a 7-fold throughput and a 15-fold space–time yield improvement (Table 3).

**Table 3:** Comparison of the batch [58] and flow conditions for the synthesis of iPrMgCl⋅LiCl.

iPrMgCl⋅LiCl	batch	flow

mmol of 2-chloropropane	100	100
[2-chloropropane] (M)	0.92	2.50
*t* (h)	12	1.50
[iPrMgCl⋅LiCl] (M)	0.89	2.10^a^
conversion of 2-chloropropane (%)	100	100
throughput (mmol⋅h^−1^)	7	50
normalized space–time yield^b^	1	15

^a^Propene and 2,3-dimethylbutane as side products. ^b^Space–time yield (mmol⋅mL^−1^⋅h^−1^): batch: 0.065, flow: 0.980.

### 4 Knochel–Hauser bases via stratified packed-bed columns containing magnesium and LiCl

**Objective:** To generate amidomagnesium lithium chloride complexes (Knochel–Hauser bases) from turbo Grignard formed in situ and the corresponding amine using stratified bicomponent packed-bed columns composed of magnesium metal and lithium chloride.

**Challenges:** Gas formation from amine deprotonation, residence time optimization due to variations in the amine and amide properties.

**System setup:** The same flow system was used as for the generation of turbo Grignard reagents (Figure S2, Supporting Information File 1). For TMPH, a coil (*V* = 10 mL, ID = 0.03″) was added downstream to increase the residence time (Figure S3, Supporting Information File 1).

We synthesized amidomagnesium chloride–lithium chloride complexes (R_2_NMgCl⋅LiCl) by the in situ formation of turbo Grignard in the presence of the corresponding amine. The reactions were carried out by flowing iPrCl/amine, 1:1 dissolved in THF/toluene, 1:1 at a 0.5 mL/min flow rate and 80 °C. Toluene was required to solubilize the magnesium amide species. During the process, propane was generated, but no overpressure was observed. The flammable gas was released after the BPR, together with the R_2_NMgCl⋅LiCl solution, in the collection flask, away from a heat source.

**System scope:** Bis(trimethylsilyl)amine (HMDS), diphenylamine (Ph_2_NH), aniline (PhNH_2_), and 2,2,6,6-tetramethylpiperidine (TMPH) were selected as substrates. Ph_2_NH, HMDS, and PhNH_2_ due to their lower p*K*_a_ (25, 30, and 31, respectively, in DMSO) [59] and TMPH due to its broad application in synthesis (Knochel–Hauser base, p*K*_a_ = 37 in DMSO) [47,59–69]. Excellent yields were obtained: HMDSMgCl⋅LiCl 1.15 M (98%), Ph_2_NMgCl⋅LiCl 1.16 M (97%), and PhNHMgCl⋅LiCl (1.15 M, 96%, Table 4, entries 1–3).

**Table 4:** Reaction between amines and isopropylmagnesium chloride generated in situ. Knochel–Hauser bases synthesis using a stratified packed-bed column of activated magnesium and lithium chloride.^a^

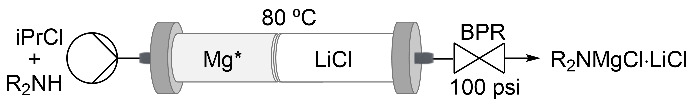				

entry	R_2_NH	[RX] (M)	[R_2_NMgX⋅LiCL] (M)	yield (%)
1	HMDS	1.2	1.17	98
2	Ph_2_NH	1.2	1.16	97
3	PhNH_2_	1.2	1.15	96

^a^A 2-chloropropane (1 equiv) and amine (1 equiv) THF/toluene, 1:1 solution (1.2 M, 30 mL) was pumped at a 0.5 mL/min flow rate and 80 °C through a column (ID = 10 mm) of activated Mg* (2 equiv) chips/powder, 1:1 and anhydrous LiCl (2 equiv) separated with fiberglass. ^b^Quantitative RX conversion. ^c^Determined by the titration of an overall R_2_MgCl·LiCl solution (≈25 mL) collected in the steady state.

**Scheme 1:**
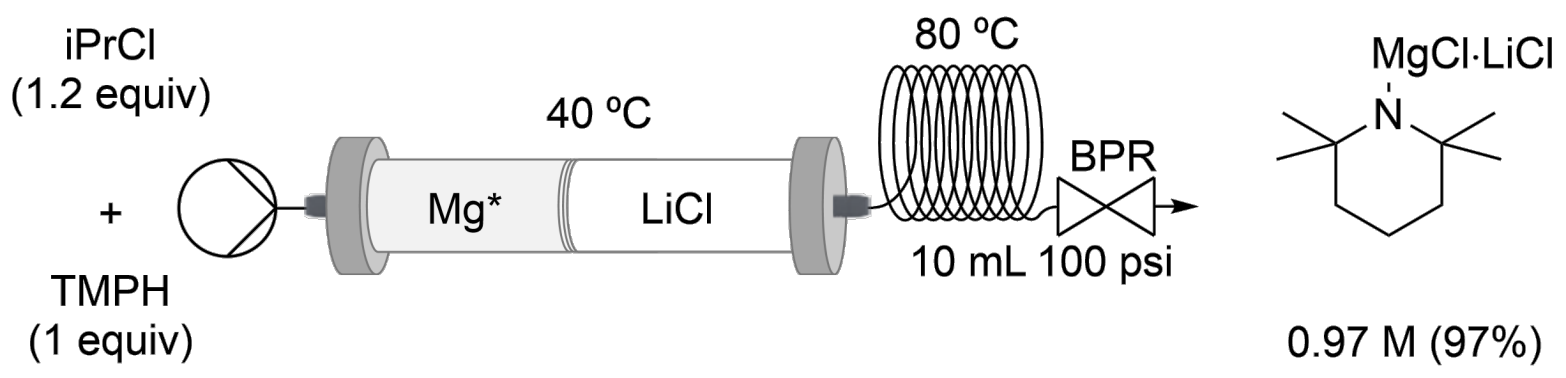
Continuous flow synthesis of TMPMgCl⋅LiCl with a stratified packed-bed column of activated magnesium and lithium chloride.

For TMPH, a 10 mL coil (20 min residence time *t*_R_) was added due to the slower reaction rate, and the iPrCl amount was optimized to 1.2 equivalents (Scheme 1). Even if the addition of a coil increased the residence time for the TMPMgCl⋅LiCl synthesis up to 25 min, our flow setup was 9 times faster than the batch version, which usually takes 24 h at room temperature [47,67]. The reaction was carried out by flowing iPrCl/TMPH, 1.2:1 dissolved in THF/toluene, 1:1 at a 0.5 mL/min flow rate and 80 °C.

As a drawback, we observed LiCl precipitation in the flask ≈2 h after collection. A clear solution can be recovered by filtration through dried fiberglass using a cannula without a drastic concentration decrease. The same reaction was done in batch using turbo Grignard generated in flow, and the same result was observed, proving that LiCl is coming from iPrMgCl⋅LiCl. The analysis of the precipitate by NMR and GC–MS, after being washed with pentane at 0 °C and dried under vacuum, showed no evidence of organic compounds. It seemed that TMPMgCl⋅LiCl coordinated less LiCl than the corresponding iPrMgCl⋅LiCl and triggered LiCl crystallization. To solve this issue, the packed-bed column temperature was decrease from 80 °C to 40 °C to reduce the amount of LiCl dissolved in the iPrMgCl⋅LiCl solution. Under these conditions, we were able to obtain TMPMgCl⋅LiCl 0.97 M (97%) as a solution (≈40 mL) that remained clear for much longer (Scheme 1). We suggest to directly react the TMPMgCl⋅LiCl solution in flow or to telescope the reagent in batch with the next step. The Knochel–Hauser base was also scaled up to ≈100 mmol using a 15 mm ID column. Using our flow procedure, a similar TMPMgCl⋅LiCl concentration (≈1.0 M) compared to the Knochel protocol was obtained [47,67], but the reaction time was reduced from 36 h to 4 h, providing a 10-fold increment in throughput and space–time yield (Table 5).

**Table 5:** Comparison between batch [47,67] and flow conditions for the synthesis of (TMPMgCl⋅LiCl).

TMPMgCl⋅LiCl	batch	flow

mmol of 2-chloropropane	100	100
[2-chloropropane] (M)	1.20	1.20
[TMPH] (M)	1.05	1.00
*t* (h)	36	4
[TMPMgCl⋅LiCl] (M)	1.03	0.97
throughput (mmol⋅h^−1^)	2	22
normalized space–time yield^a^	1	10

^a^Space–time yield (mmol⋅mL^−^1⋅h^−1^): batch: 0.016, flow: 0.160.

**Scheme 2:**
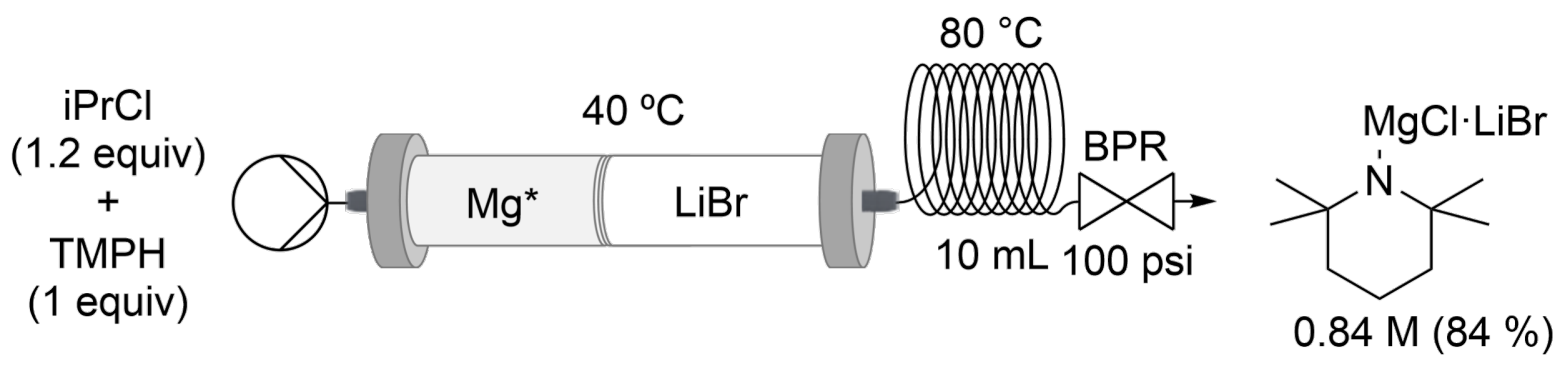
Continuous flow synthesis of TMPMgCl⋅LiBr with a stratified packed-bed column of activated magnesium and lithium bromide.

We also found that LiBr could be used. The reaction was carried out under the same conditions. The high solubility of LiBr provided solutions that remained clear for days (TMPMgCl⋅LiBr 0.84 M, 84%, Scheme 2).

### 5 Alkoxide bases via stratified packed-bed columns containing magnesium and LiCl

**Objective:** To generate magnesium alkoxide–lithium chloride complexes by telescoped reactions of turbo Grignards with *tert*-amyl alcohol using stratified bicomponent packed-bed columns composed of magnesium metal and lithium chloride, a T-mixer, and a coil reactor.

**Challenges:** Alcohol incompatibility with the activating solution and alkoxide solubility.

**System setup:** The same flow system was used as for the generation of turbo Grignard reagents. Extra feed was added between the packed-bed column and the coil (*V* = 10 mL, ID = 0.03″) for the *tert*-amyl alcohol addition (Figure S4, Supporting Information File 1).

**Scheme 3:**
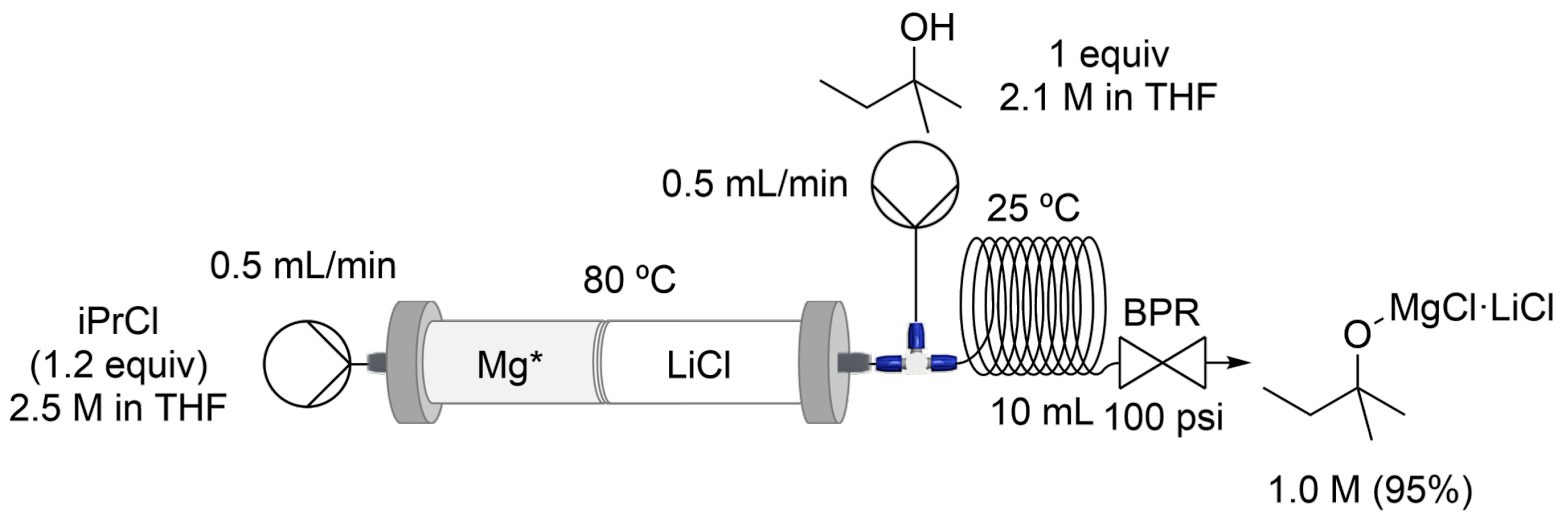
Continuous flow synthesis of *t*-AmylOMgCl⋅LiCl with a stratified packed-bed column of activated magnesium and lithium chloride.

Finally, we explored the formation of sterically hindered oxygen bases by a direct alcohol deprotonation. Knochel-type *tert*-amyl magnesium alkoxide (*t*-AmylOMgCl⋅LiCl) 1.0 M (95%) was obtained (≈15 mL) by the reaction of the corresponding alcohol (1.0 equiv) and turbo Grignard (1.2 equiv) under flow conditions at 25 °C (Scheme 3). For *t*-AmylOMgCl⋅LiCl, the concentration was determined using a mixture of benzoic acid and thymolphthalein as an indicator [70].

### 6 On-demand reagent proof of concept

**Objective:** To reiterate the turbo Grignard and Knochel–Hauser base synthesis on the ODR prototype.

**Challenges:** The changes in the reactor material from reusable glass to disposable perfluorinated columns and the modification in the bicomponent (Mg/LiCl) configuration from a single stratified column to two separated monocomponent columns.

**System setup:** The internal diameter of the perfluorinated tubular reactor used on the ODR prototype was limited to 6.3 mm to maintain an efficient heat transfer. Due to this ID limitation and the heater dimensions, we decided to separate Mg and LiCl in two tubular reactors.

**Figure 6:**
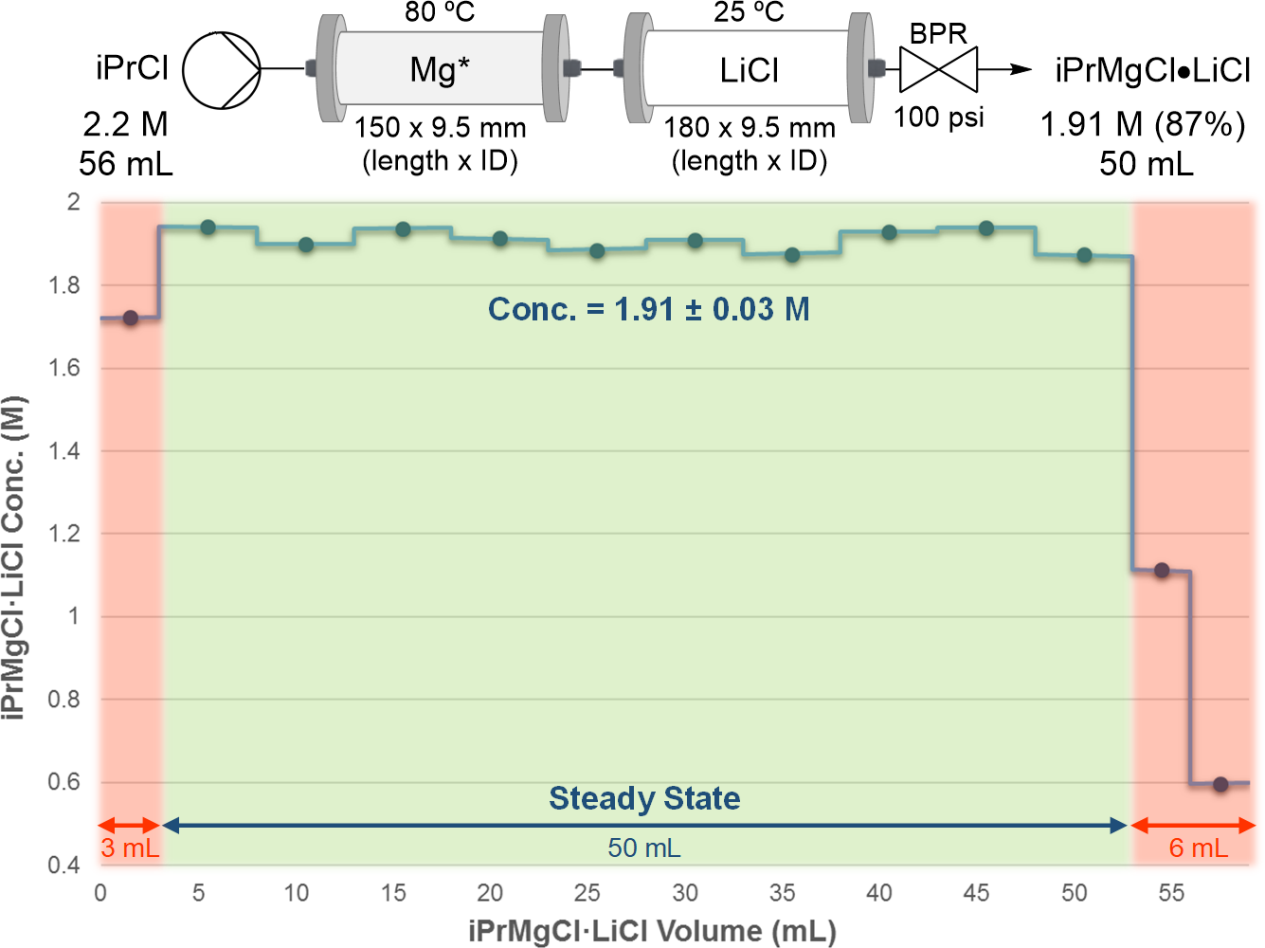
Steady-state concentration stability during the conversion of iPrCl in THF (56 mL, 2.2 M) into iPrMgCl·LiCl (50 mL, 1.91 M) using two perfluorinated disposable monocomponent tubular reactors.

First, the concentration stability in the steady state and the scalability up to ≈100 mmol was verified using two perfluorinated tubular reactors of 9.5 mm (ID) on the Vapourtec flow system. The first column containing Mg was heated at 80 °C, using a temperature-controlled glass manifold [51]. The LiCl column was kept at 25 °C. The concentration was followed over time during the conversion of 2-chloropropane in THF (56 mL, 2.2 M) into iPrMgCl·LiCl (50 mL, 1.91 M, Figure 6). Ten samples of 5 mL were collected, and the concentration was determined in duplicates using 2-hydroxybenzaldehyde phenylhydrazone.

The results demonstrated a continuous and stable generation of iPrMgCl⋅LiCl (≈100 mmol) in the steady state under similar ODR prototype conditions. A certain volume of the starting material solution (6 mL) was discarded to prevent the dilution of iPrMgCl⋅LiCl at the beginning and at the end of the experiment due to solvent diffusion. We stipulate that these results illustrate that our system provides a high-quality material for the discovery-scale needs. This approach is not suitable for a large scale and is not designed to be scaled. The goal is to aid discovery efforts to increase the reagent reliability.

**Scheme 4:**
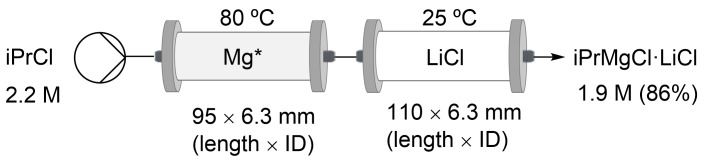
Synthesis of iPrMgCl⋅LiCl on the ODR prototype.

The synthesis of ≈10 mL turbo Grignard, iPrMgCl⋅LiCl, and ≈10 mL of the Knochel–Hauser base derived from HMDS, HMDSMgCl⋅LiCl, was reiterated on the ODR prototype. Similarly, an iPrMgCl⋅LiCl yield of 86% was obtained (Scheme 4) using the optimized conditions established on a commercial flow system with a single reusable bicomponent glass column (Table 2, entry 2) and with two separated disposable monocomponent columns (Figure 6). The cartridge is composed of three solution bags (THF, the activating solution, and the iPrCl solution) and two tubular reactors (Mg chips/powder and LiCl) connected in-series (Figure S5, Supporting Information File 1).

**Scheme 5:**
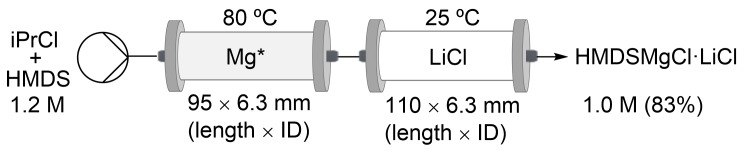
Synthesis of HMDSMgCl⋅LiCl on the ODR prototype.

For HMDSMgCl⋅LiCl, the same cartridge configuration was used (Figure S5, Supporting Information File 1), and a slightly lower yield (83%) was obtained (Scheme 5) compared to the reaction done on the Vapourtec flow system (Table 4, entry 1). This variation was attribute to the unsteady flow rate produced by propane released during the reaction, and thus affecting the fluid dynamics and the back-pressure control.

The product purities, a quantitative iPrCl conversion, and the yields were confirmed by NMR (Section 5, Supporting Information File 1), demonstrating the ODR prototype ability to safely produce high-quality organomagnesium reagents on demand.

## Conclusion

We have developed a new flow setup for the on-demand synthesis of highly concentrated (≈2 M) turbo Grignards from alkyl chlorides using a stratified packed-bed column of activated magnesium and lithium chloride. The volumes we can produce reliably are suitable for the target discovery scale audience. The magnesium activation in a packed-bed column is safer and faster in comparison to batch protocols. LiCl enhances the solubility and reactivity of organomagnesium compounds, and our moisture-free setup makes it possible to directly use the hygroscopic LiCl in a solid form. The back-pressure control allows a high-temperature oxidative addition reaction and enables the quantitative conversion of less reactive but more cost-effective alkyl chlorides. Furthermore, a low-cost pod-style synthesizer prototype has been designed and built. The reagents were prepacked in disposable perfluorinated assemblies–bags, cartridges, and tubings–sealed together using a new thermal bonding method. This on-demand concept was demonstrated by preparing the turbo Grignard reagent and the Knochel–Hauser base (optimized on a commercial flow system). We predict that with small modifications, this system could be configured to produce many different reagents. Our group is currently working on an organolithium version of this on-demand reagent approach.

## Experimental

**Turbo Grignard: isopropylmagnesium chloride–lithium chloride complex (iPrMgCl⋅LiCl):** 2-Chloropropane (2.975 g, 3.46 mL, 37.5 mmol, 1 equiv) was dissolved in THF (11.5 mL) in a flask under argon. A 2.5 M organic-halide solution was flowed through a column (ID = 10 mm, length = 100 mm) of activated magnesium (chips/powder, 1:1, w/w, 1.86 g, 75 mmol, 2 equiv) and anhydrous lithium chloride (3.21 g, 75 mmol, 2 equiv), with a BPR (100 psi) at a 0.5 mL/min flow rate and at 80 °C. After ≈4 min, the outcome solution was collected in a vial under an inert atmosphere containing 2-hydroxybenzaldehyde phenylhydrazone (20–40 mg). When the yellow-colored solution turned orange, the turbo Grignard reagent was collected in a flask under argon. When the starting material solution ran out, the organomagnesium collection was maintained for 4 min (≈2-fold the residence time), flowing THF at 0.5 mL/min, yielding 88% of the isopropylmagnesium chloride–lithium chloride complex as clear 2.19 M solution (≈10 mL).

**Knochel**–**Hauser base: lithium dichloro(2,2,6,6-tetramethylpiperidinato)magnesate (TMPMgCl⋅LiCl):** 2-Chloropropane (4.284 mg, 4.99 mL, 54.0 mmol, 1.2 equiv) and 2,2,6,6-tetramethylpiperidine (TMPH, 6.420 g, 7.67 mL, 45.0 mmol, 1.0 equiv) were dissolved in THF (16.2 mL) and toluene (16.2 mL) in a flask under argon. The mixed solution of the organic halide (1.2 M) and the amine was flowed through a column (ID = 10 mm, length = 100 mm) of activated magnesium (chips/powder, 1:1, w/w, 2.23 g, 90 mmol, 2 equiv) and lithium chloride (3.85 g, 90 mmol, 2 equiv) at 0.5 mL/min, 40 °C, and atmospheric back pressure. After ≈4 min, the outcome solution was collected in a vial under an inert atmosphere containing 2-hydroxybenzaldehyde phenylhydrazone (20–40 mg). When the yellow-colored solution turned orange, the mixture was flowed through the coil at 0.5 mL/min, 80 °C, and 100 psi back pressure. When the starting material solution ran out, THF/toluene, 1:1 was pumped at 0.5 mL/min to maintain the mixture flowing. After ≈20 min, the gas released was observed, and the outcome solution was collected in a vial under an inert atmosphere containing 2-hydroxybenzaldehyde phenylhydrazone (20–40 mg). When the yellow-colored solution turned orange, the Knochel–Hauser base (TMPMgCl⋅LiCl) solution was collected in a flask under argon. The organomagnesium collection was maintained for 20 min or until the gas release started to decrease, yielding 97% of the 2,2,6,6-tetramethylpiperidinylmagnesium chloride–lithium chloride complex (TMPMgCl⋅LiCl) solution as clear 0.97 M solution (≈40 mL).

**Knochel-type magnesium alkoxide (*tert*-amylOMgCl⋅LiCl):** 2-Chloropropane (1.983 g, 2.31 mL, 25.0 mmol, 1.2 equiv) was dissolved in THF (7.7 mL) in a flask under argon. 2-Methyl-2-butanol (1.87 g, 2.32 mL, 21.0 mmol, 1.0 equiv) was dissolved in THF (7.7 mL) in a second flask under argon. The 2.5 M organic-halide solution was flowed through a column (ID = 10 mm, length = 100 mm) of activated magnesium (chips/powder, 1:1, w/w, 1.49 g, 60 mmol, 2.4 equiv) and lithium chloride (2.57 g, 60 mmol, 2.4 equiv) at 0.5 mL/min, 80 °C, and 100 psi back pressure. After ≈4 min, the outcome solution was collected in a vial under an inert atmosphere.

## Supporting Information

All details for the flow procedures and reactors assembly (full part list, flow system photos, and ODR prototype protocols) and all experimental data of the chemical reactions (optimization, packed-bed particle size study, and concentration determination) and NMR spectra.

File 1 Additional experimental data.
