# Supplementary material for: Disposable cartridge concept for the on-demand synthesis of turbo Grignards, Knochel–Hauser amides, and magnesium alkoxides
Source: Beilstein J Org Chem. 2020 Jun 19;16:1343–56. doi: 10.3762/bjoc.16.115 (PMC7308606; doi:10.3762/bjoc.16.115)

BEILSTEIN JOURNAL OF ORGANIC CHEMISTRY

Supporting Information

for

Disposable cartridge concept for the on-demand synthesis of turbo Grignards, Knochel–Hauser amides, and magnesium alkoxides

Mateo Berton, Kevin Sheehan, Andrea Adamo and D. Tyler McQuade

Beilstein J. Org. Chem. **2020,** *16,* 1343–1356. doi:10.3762/bjoc.16.115

Additional experimental data

License and Terms: This is a supporting information file under the terms of the Creative Commons Attribution License (http://creativecommons.org/licenses/by/4.0). Please note that the reuse, redistribution and reproduction in particular requires that the authors and source are credited.
The license is subject to the *Beilstein Journal of Organic Chemistry* terms and conditions: (https://www.beilstein-journals.org/bjoc)

Table of contents

page

1. Experimental procedures
   1.1. General S2
   1.2. General flow procedures
      1.2.1. Assembly and setup of the packed-bed column reactor S2
      1.2.2. General flow procedure for the magnesium activation S2
      1.2.3. Grignard reagents (RMgX) S3
      1.2.4. Turbo Grignard reagents (RMgX·LiCl) S4
      1.2.5. Amidomagnesium chloride lithium chloride complexes derived from HMDS (HMDSMgCl·LiCl), diphenylamine (Ph_2NMgCl·LiCl), and aniline (PhNHMgCl·LiCl) S4
      1.2.6. Knochel–Hauser base: lithium dichloro(2,2,6,6-tetramethylpiperidinato)magnesate (TMPMgCl·LiCl) S5
      1.2.7. Lithium bromochloro(2,2,6,6-tetramethylpiperidinato)magnesate (TMPMgCl·LiBr) S6
      1.2.8. Knochel-type magnesium alkoxide (*tert*-amylOMgCl·LiCl) S6
      1.2.9. Determination of the basicity concentration
         1.2.9.1. C–Mg bases S7
         1.2.9.2. N–Mg bases S7
         1.2.9.3. O–Mg bases S7
      1.2.10. NMR titration with 1,5-cyclooctadiene as a standard S7
   1.3. ODR prototype procedures S7
      1.3.1. Turbo Grignard (iPrMgCl·LiCl) S9
      1.3.2. Amidomagnesium chloride lithium chloride complex derived from HMDS (HMDSMgCl·LiCl) S8
2. Results and discusion
   2.1. Grignard reagents via magnesium packed beds S8
   2.2. Turbo Grignards via stratified packed-bed columns containing magnesium and LiCl S11
3. References S12
4. Author contributions S12
5. NMR spectra
   5.1. Titration of turbo Grignard (iPrMgCl·LiCl) in THF with COD in toluene-d_8 S13
   5.2. Titration of the HMDS amidomagnesium chloride LiCl complex (HMDSMgCl·LiCl) in THF/toluene with COD in toluene-d_8 S14
   5.3. Turbo Grignard (iPrMgCl·LiCl) generated with ODR prototype in THF with COD in toluene-d_8 S15
   5.4. HMDS amidomagnesium chloride LiCl complex (HMDSMgCl·LiCl) in THF/toluene generated with the ODR prototype with COD in toluene-d_8 S16

1. Experimental procedures

1.1. General

Drisolv THF, toluene, and anhydrous Et_2O (in a septum-sealed bottle) as well as anhydrous 2-methyltetrahydrofuran (≥99%, inhibitor-free, sure/seal) were purchased from Sigma-Aldrich. Mg powder (20–230 mesh, 98%), Mg chips (6–35 mesh, 99.98%), anhydrous LiBr (Redi-Dri, ≥99%), 2-bromopropane (≥99%), bromoethane (≥99%), 1-bromooctane (≥99%), 2-chlorobutane (≥99%), 1-chlorobutane (≥99%), anhydrous chlorobenzene (99.8%), benzyl chloride (99%), iodomethane (≥99%), diphenylamine (≥99%), HMDS (98%), TMPH (≥99%), anhydrous 2-methyl-2-butanol (≥99%), 2-hydroxybenzaldehyde phenylhydrazone (97%), benzoic acid (≥99.9%), COD (sure/seal, ≥99%), DIBAL-H (1.0 M in toluene), and lithium chloride (0.5 M in anhydrous THF) were purchased from Sigma-Aldrich and used without further purification. Anhydrous lithium chloride (≥99%) was purchased from Bio Basic and stored in a desiccator cabinet. 1-Bromo-2-chloroethane (>98%), TMSCl (>98%), 2-chloropropane (>99%), 4-(phenylazo)diphenylamine (≥98%), and thymolphthalein were purchased from TCI Chemicals and used as received.

1.2. General flow procedures

A commercial flow chemistry system (Vapourtec R-Series)[1] is used, equipped with:

- Temperature controlled glass manifold reactor (10 –15 mm bore columns)–catalog# 50-1013
- Standard tube reactor manifold–catalog# 50-1009
- Stainless steel reactor coil (10 mL)–catalog# 50-1234

Diba Omnifit EZ SolventPlus[2] columns with 1 fixed and 1 adjustable end-piece are used to prepare packed bed reactors:

- 10 × 100 mm (catalog# 006EZS-10-10-AF)
- 15 × 100 mm (catalog# 006EZS-15-10-AF)
- PTFE frits 10 µm

Pumping is carried out using either HPLC pumps from Vapourtec system or Chemyx Fusion 4000/6000 syringe pumps and Harvard stainless steel syringes with Chemraz perfluoroelastomer O-rings.

Pressure is controlled by Idex back pressure regulators (BPR)–100 psi (7.0 bar), check valves and shut-off valves.

We have found that both glass and perfluoroalkoxy alkane (PFA) columns with similar dimensions can be used. To reduce the costs, the flow chemistry system can be replaced by syringe or HPLC pumps and reactor heating can be accomplished using standard heating tools (water/oil bath, heating jacket or suitable oven).

1.2.1. Assembly and setup of the packed-bed column reactor

Vacuum grease is put on the O-rings of both Omnifit column end-pieces. The lower end piece, with its corresponding frit, is fitted to one end of the glass column and the retaining nut is screwed onto the glass threads. A mixture of Mg chips/powder 1:1 wt % is loaded in the column. The adjustable upper end-piece (plunger), with its corresponding frit, is screwed to the retaining nut. The plunger is adjusted until no empty gap is observed. Mg must stay loose to avoid channeling. The column reactor is inserted into the glass heat exchanger manifold. Inlet and outlet PFA tubing are respectively attached to the lower and upper end-piece connection caps. A back pressure regulator is attached to the outlet. The entire setup is placed on the reactor module and the temperature sensor is connected (Figure S1).

In the case of the Mg/LiCl bicomponent column, Mg (chips/powder) is first loaded, followed by a piece of fiber glass (stored in the oven at 120 °C) and finally LiCl. Lithium chloride loading and subsequent plunger assembly have to be done as quick as possible or in glovebox to limit moisture uptake.

The column is primed with anhydrous solvent for 5 min at flow rate of 1 mL/min.

1.2.2. General flow procedure for the magnesium activation[3]

After priming the column with anhydrous THF, the magnesium is activated using a single solution containing 1-bromo-2-chloroethane (345 mg, 0.2 mL, 2.4 mmol), chlorotrimethylsilane (TMSCl, 685 mg, 0.8 mL, 6.2 mmol), THF (4.5 mL) and DIBAL-H 1.0 M in toluene (4.5 mL, 4.5 mmol) prepared in a flask under an inert atmosphere. The activating solution is flowed through Mg column with BPR (pressure depending reaction requirement) at 1 mL/min and 25 °C. Gas released (acetylene) indicates activation is taking place.

Same activation protocol for Mg/LiCl bicomponent column.

When longer residence time is needed or the addition of a substrate is required, a 10 mL stainless steel coil (ID = 0.03 inch) is connected after the packed bed reactor. In this cases, the activating solution outcome is discarded before entering the coil to avoid clogging.

Best results are obtained when organohalide solution are flowed immediately after activation. No washing step needed.

1.2.3. Grignard reagents (RMgX)

Bromoethane (3.336 g, 2.28 mL, 30.00 mmol, 1 equiv) is dissolved in THF (22.7 mL) in a flask under argon. The organohalide 1.2 M solution is flowed through activated magnesium (chips/powder 1:1 wt %, 1.49 g, 60 mmol, 2 equiv) with BPR (100 psi) at 0.5 mL/min and 25 °C (Figure S1). After ≈4 min, the outcome solution is collected in a vial containing 2-hydroxybenzaldehyde phenylhydrazone (20–40 mg). When the yellow color solution turns orange, Grignard reagent solution is collected in a flask under argon. When the starting material solution is consumed, organomagnesium collection is maintained during 4 min (≈2-fold the residence time), pumping THF at 0.5 mL/min, yielding 90% of ethylmagnesium bromide as clear 1.08 M solution (≈20 mL, the yield is calculated in a steady state because the discarded volume of head and tail solution is constant, independently of the volume of organohalide solution converted). The concentration determination with 2-hydroxybenzaldehyde phenylhydrazone (82.3 mg, 0.38 mmol) dissolved in THF (1 mL) consumed 0.37 mL of EtMgBr corresponding to 1.03 M. The concentration determination with benzoic acid (46.2 mg, 0.37 mmol) and 4-(phenylazo)diphenylamine (indicator) dissolved in THF (1 mL) consumed 0.33 mL of EtMgBr corresponding to 1.12 M.

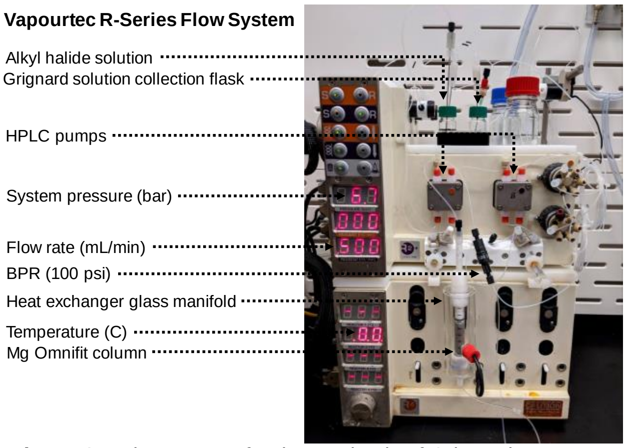

Figure S1. Flow system for the synthesis of Grignard reagents.

For the reaction at higher temperature, the heat exchanger is turned on after activation (remaining activating solution presents in the column). Once the target temperature is attained, the organohalide solution is pumped through the activated magnesium. The concentration determination results of organomagnesium reagents (Table S1).

Table S1. Concentration determination of the organomagnesium reagents.[4,5]

Grignard compound	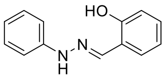 (mg)	RMgX *V* (mL)	[RMgX] (M)	$PhCO_2H$ (mg) + 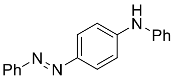	RMgX *V* (mL)	[RMgX] (M)	average *c* (M)	approx. collected volume (mL)	yield[a] (%)
iPrMgBr	71.0	0.45	0.73	48.1	0.52	0.75	0.74	20	82
EtMgBr	82.3	0.37	1.02	46.2	0.33	1.13	1.08	20	90
EtMgBr (2-MeTHF)	100.1	0.21	2.18	63.2	0.23	2.23	2.21	15	88
EtMgBr (Et_2O)	93.7	0.17	2.38	94.1	0.32	2.39	2.39	15	96
n-OctMgBr	80.7	0.72	0.51	23.7	0.38	0.51	0.51	30	85
n-OctMgBr (Et_2O)	88.5	0.37	1.09	57.2	0.40	1.07	1.08	20	90
iPrMgCl	99.8	0.19	2.40	46.6	0.16	2.36	2.38	15	95
sec-BuMgCl	55.7	0.12	2.12	60.6	0.21	2.34	2.23	15	89
n-BuMgCl	113.9	0.22	2.37	58.4	0.20	2.37	2.37	15	95
PhMgCl[b]	107.3	0.21	2.34	90.8	0.32	2.30	2.32	15	93
BnMgCl[c]	83.6	0.39	0.98	68.5	0.56	0.99	0.99	20	83
MeI (Et_2O)[d]	129.1	0.25	2.36	79.3	- [e]	- [e]	2.36	15	94

[a] Yield in a steady state. [b] 100 °C. [c] 2-MeTHF/THF (9:1) and 60 °C. [d] 25 °C and 140 psi BPR. [e] Turned purple immediately.

1.2.4. Turbo Grignard reagents (RMgX·LiCl)

2-Chloropropane (2.975 g, 3.46 mL, 37.5 mmol, 1 equiv) is dissolved in THF (11.5 mL) in a flask under argon. The organohalide 2.5 M solution is flowed through activated magnesium (chips/powder 1:1 wt %, 1.86 g, 75 mmol, 2 equiv) and anhydrous lithium chloride (3.21 g, 75 mmol, 2 equiv) with BPR (100 psi) at 0.5 mL/min and 80 °C (Figure S2). After ≈4 min, the outcome solution is collected in a vial containing 2-hydroxybenzaldehyde phenylhydrazone (20–40 mg). When the yellow color solution turns orange, the turbo Grignard reagent is collected in a flask under argon. When the starting material solution is consumed, the organomagnesium collection is maintained during 4 min (≈2-fold the residence time), flowing THF at 0.5 mL/min. Yielding 88% of isopropylmagnesium chloride lithium chloride complex as clear 2.19 M solution (≈10 mL, darker than the corresponding Grignard reagent, yield is calculated in a steady state because the discarded volume of head and tail solution is constant, independently of the volume of organohalide solution converted). The concentration determination with 2-hydroxybenzaldehyde phenylhydrazone (121.5 mg, 0.56 mmol) dissolved in THF (1 mL) consumed 0.25 mL of iPrMgCl·LiCl corresponding to 2.24 M. The concentration determination with benzoic acid (47.1 mg, 0.39 mmol) and 4-(phenylazo)diphenylamine (indicator) dissolved in THF (1 mL) consumed 0.18 mL of iPrMgCl·LiCl corresponding to 2.14 M. NMR titration was done diluting *i*-PrMgCl·LiCl solution (0.6 mL) in toluene-d_8 (0.3 mL) with 1,5-cyclooctadiene (COD, 43.0 mg, 0.394 mmol) as standard (NMR spectra in Section 5.1).[6]

$$c = \frac{0.394\ \text{mmol}}{0.6\ \text{mL}} \times \frac{3.49 \times 4\text{H}}{4 \times 1\text{H}} = 2.29\ \text{M}$$

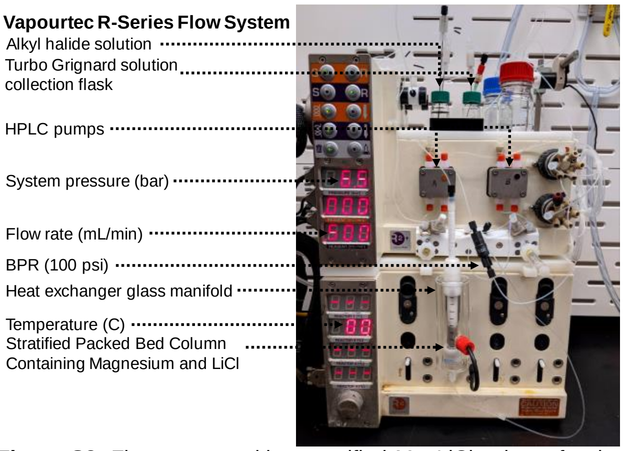

Figure S2. Flow system with a stratified Mg–LiCl column for the synthesis of turbo Grignard reagents and Knochel–Hauser bases derived from HMDS, Ph_2NH, and $PhNH_2$.

The concentration determination results of turbo Grignard reagents:

Table S2. Concentration determination of turbo Grignard reagents.[4,5]

turbo Grignard compound	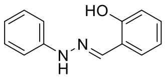 (mg)	RMgX *V* (mL)	[RMgX] (M)	$PhCO_2H$ (mg) + 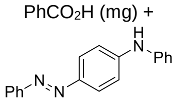	RMgX *V* (mL)	[RMgX] (M)	average *c* (M)	approx. collected volume (mL)	yield[a] (%)
EtMgBr·LiCl	76.9	0.26	1.37	34.6	0.23	1.22	1.30	20	87
iPrMgCl·LiCl	121.5	0.25	2.24	121.5	0.18	2.14	2.19	10	88
sec-BuMgCl·LiCl	106.4	0.22	2.21	74.8	0.29	2.09	2.15	10	86
n-BuMgCl·LiCl	154.5	0.33	2.14	39.2	0.15	2.12	2.13	10	85

[a] Yield in a steady state

1.2.5. Amidomagnesium chloride lithium chloride complexes derived from HMDS (HMDSMgCl·LiCl), diphenylamine (Ph_2NMgCl·LiCl), and aniline (PhNHMgCl·LiCl)

2-Chloropropane (2.975 g, 3.46 mL, 37.5 mmol, 1 equiv) and bis(trimethylsilyl)amine (5.601 g, 7.83 mL, 37.5 mmol, 1 equiv) are dissolved in THF (10.0 mL) and toluene (10.0 mL) in a flask under an inert atmosphere. The organohalide and amine 1.2 M solution is flowed through activated magnesium (chips/powder 1:1 wt %, 1.86 g, 75 mmol, 2 equiv) and anhydrous lithium chloride (3.21 g, 75 mmol, 2 equiv) with BPR (100 psi) at 0.5 mL/min and 80 °C. The same conditions and procedure as for turbo Grignard reagents are used flowing THF/toluene (1:1) as solvent. Yielding 98% of bis(trimethylsilyl)amidomagnesium chloride lithium chloride complex as clear 1.17 M solution (≈25 mL, yield is calculated in a steady state because the discarded volume of head and tail solution is constant, independently of the volume of organohalide solution converted). The concentration determination with 2-hydroxybenzaldehyde phenylhydrazone (133.2 mg, 0.61 mmol) dissolved in THF (1 mL) consumed 0.52 mL of HMDSMgCl·LiCl corresponding to 1.17 M. The concentration determination with benzoic acid (30.3 mg, 0.25 mmol) and thymolphthalein (indicator) dissolved in THF (1 mL) consumed 0.21 mL of HMDSMgCl·LiCl corresponding to 1.17 M. The NMR titration was done diluting HMDSMgCl·LiCl solution (0.6 mL) in toluene-d_8 (0.3 mL) with 1,5-cyclooctadiene (COD, 36.9 mg, 0.338 mmol) as standard (NMR spectra in Section 5.2).[6]

$$c = \frac{0.338\ \mathrm{mmol}}{0.6\ \mathrm{mL}} \times \frac{32.22 \times 4\mathrm{H}}{4 \times 18\mathrm{H}} = 1.01\ \mathrm{M}$$

The concentration determination results of amidomagnesium chloride LiCl reagents:

Table S3. Concentration determination of amidomagnesium chloride LiCl reagents.[4]

amidomagnesium chloride·LiCl reagent	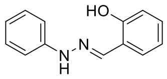 (mg)	RMgX *V* (mL)	[RMgX]. (M)	$PhCO_2H$ (mg) + thymolphthalein	RMgX *V* (mL)	[RMgX] (M)	average *c* (M)	approx. collected volume (mL)	yield[a] (%)
HMDSMgCl·LiCl	133.2	0.54	1.13	37.3	0.25	1.21	1.17	25	98
Ph_2NMgCl·LiCl	98.6	0.40	1.13	25.0	0.17	1.19	1.16	25	97
PhNHMgCl·LiCl	56.8	0.40	1.15	70.0	0.28	1.14	1.15	25	96

[a] Yield in a steady state

The benzoic acid and 4-(phenylazo)diphenylamine mixture can be used to confirm the absence of unreacted turbo Grignard.

1.2.6. Knochel–Hauser base: lithium dichloro(2,2,6,6-tetramethylpiperidinato)magnesate (TMPMgCl·LiCl)

The set-up consists of the stratified bicomponent packed bed reactor, a shut-off valve, a 10 mL stainless steel coil reactor and a BPR (100 psi, Scheme S1). All the system is primed with THF/toluene with the coil heated at 80 °C. Then the shut-off valve is closed and activation is done at atmospheric pressure. Activating solution outcome is discarded before entering to coil to avoid clogging.

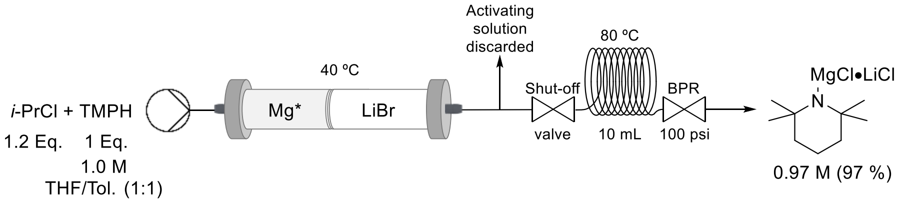

Scheme S1. Schematic plug flow setup for the synthesis of TMPMgCl·LiCl.

2-Chloropropane (4.284 mg, 4.99 mL, 54.0 mmol, 1.2 equiv) and 2,2,6,6-tetramethylpiperidine (TMPH, 6.420 g, 7.67 mL, 45.0 mmol, 1.0 equiv) are dissolved in THF (16.2 mL) and toluene (16.2 mL) in a flask under an inert atmosphere. The organohalide 1.2 M and amine 1.0 M mixed solution is flowed through activated magnesium (chips/powder 1:1 wt %, 2.23 g, 90 mmol, 2 equiv) and lithium chloride (3.85 g, 90 mmol, 2 equiv) at 0.5 mL/min, 40 °C and atmospheric back pressure. After ≈4 min, the outcome solution is collected in a vial under an inert atmosphere containing 2-hydroxybenzaldehyde phenylhydrazone (20–40 mg). When the yellow color solution turns orange, the mixture is flowed through the coil at 0.5 mL/min, 80 °C and 100 psi back pressure (Figure S3). When the starting material solution is consumed, THF/toluene (1:1) is pumped at 0.5 mL/min to maintain the mixture flowing. After ≈20 min, gas released (mostly propane) is observed and the outcome solution is collected in a vial containing 2-hydroxybenzaldehyde phenylhydrazone (20–40 mg). When the yellow color solution turns orange, the Knochel–Hauser base (TMPMgCl·LiCl) solution is collected in a flask under argon. The organomagnesium collection is maintained during 20 min or until gas release starts to decrease. Yielding 97% of 2,2,6,6-tetramethylpiperidinylmagnesium chloride lithium chloride complex (TMPMgCl·LiCl) solution as clear 0.97 M solution (≈40 mL, yield is calculated in a steady state because the discarded volume of head and tail solution is constant, independently of the volume of organohalide solution converted). The concentration determination (duplicate) with 2-hydroxybenzaldehyde phenylhydrazone (121.5 mg, 0.56 mmol and 85.5 mg, 0.39 mmol) dissolved in THF (1 mL) consumed 0.25 mL and 0.18 ml of TMPMgCl·LiCl corresponding to 0.95 M and 0.99 M respectively.

A benzoic acid and 4-(phenylazo)diphenylamine mixture or thymolphthalein can be used to confirm the absence of unreacted turbo Grignard.

The sampling for the concentration determination eventually triggers LiCl precipitation. Solid filtration through fiber glass by cannula yields to similar concentration solution. Precipitation issue can be avoided using this reagent in flow or to telescope the reagent in batch with next step reaction mixture.

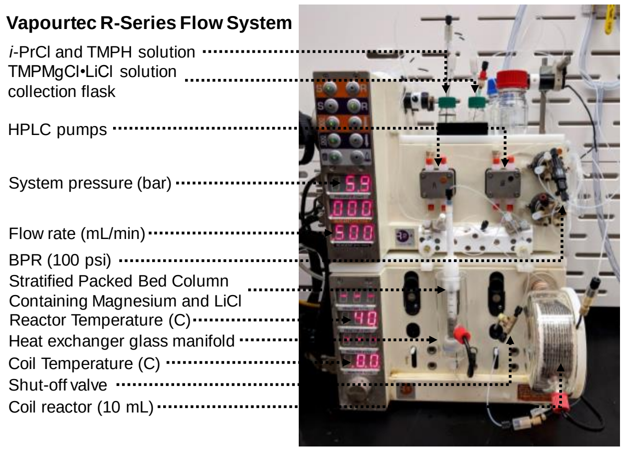

Figure S3. Flow chemistry system for the synthesis of Knochel–Hauser base (TMPMgCl·LiCl).

1.2.7. Lithium bromochloro(2,2,6,6-tetramethylpiperidinato)magnesate (TMPMgCl · LiBr)

Same conditions and procedure as for Knochel–Hauser base (TMPMgCl·LiCl) are used. LiCl is replaced by LiBr (3.95 g, 45 mmol, 2 equiv). Due to LiBr higher molecular weight and limited volume of the column, the amount of 2,2,6,6-tetramethylpiperidine (TMPH) converted is reduced to 22.5 mmol.

1.2.8. Knochel-type magnesium alkoxide (*tert*-amylOMgCl·LiCl)

Same set-up as for Knochel–Hauser base synthesis is used with the following exceptions: the stratified bicomponent column is heated at 80 °C with a 100 psi BPR, a T-union is added between packed bed and coil reactors for alcohol addition and coil reactor is maintained at 25 °C (Scheme S2). Syringe pump is employed for better control of *tert*-amylOH addition.

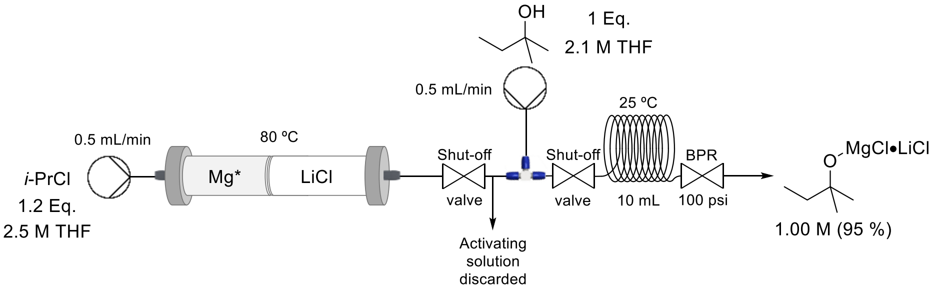

Scheme S2. Schematic plug flow setup for the synthesis of *tert*-amylOMgCl·LiCl.

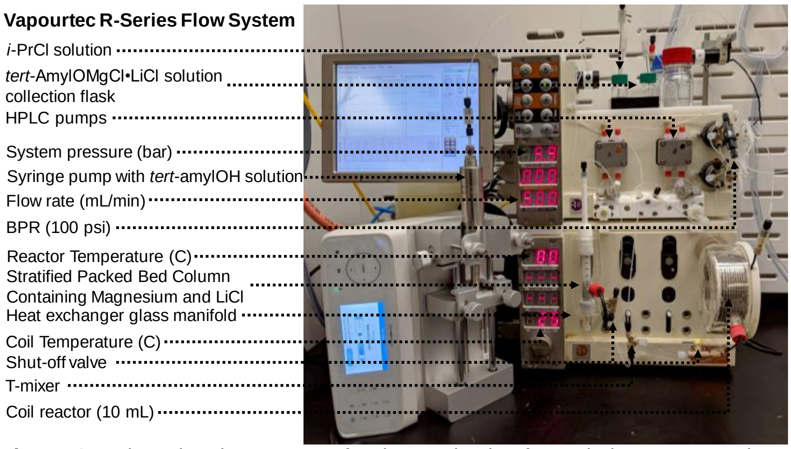

Figure S4. Flow chemistry system for the synthesis of Knochel-type magnesium alkoxide (*tert*-amylOMgCl·LiCl).

2-Chloropropane (1.983 g, 2.31 mL, 25.00 mmol, 1.2 equiv) is dissolved in THF (7.7 mL) in a flask under an inert atmosphere. 2-Methyl-2-butanol (1.87 g, 2.32 mL, 21.0 mmol, 1.0 equiv) is dissolved in THF (7.7 mL) in a second flask under an inert atmosphere. The organohalide 2.5 M solution is flowed through activated magnesium (chips/powder 1:1 wt %, 1.49 g, 60 mmol, 2.4 equiv) and lithium chloride (2.57 g, 60 mmol, 2.4 equiv) at 0.5 mL/min, 80 °C and 100 psi back pressure (after first shut-off valve). After ≈4 min, the

outcome solution is collected in a vial under an inert atmosphere containing 2-hydroxybenzaldehyde phenylhydrazone (20–40 mg). When the yellow color solution turns orange, Grignard reagent solution and 2-methyl-2-butanol solution are pumped together through a T-union at 0.5 mL/min each feed. The mixture is flowed through the coil at 1.0 mL/min, 25 °C and 100 psi back pressure (Figure S4). After ≈10 min, gas released (mostly propane) is observed and the outcome solution is collected in a vial under an inert atmosphere containing benzoic acid (10–20 mg) and thymolphthalein (indicator). When the colorless solution turns blue, the magnesium alkoxide base solution is collected in a flask under argon. When the starting material solutions are consumed, THF is pumped at 0.5 mL/min per each feed to maintain the mixture flowing at the same flow rate. The organomagnesium collection is maintained during 10 min or until gas release starts to decrease. Yielding 95% of magnesium *tert*-amyloxide chloride lithium chloride complex (*tert*-amylOMgCl·LiCl) as clear 1.00 M solution (≈15 mL, yield is calculated in a steady state because the discarded volume of head and tail solution is constant, independently of the volume of organohalide solution converted). The concentration determination (duplicate) with benzoic acid (30.9 mg, 0.25 mmol and 40.7 mg, 0.33 mmol) and thymolphthalein (indicator) dissolved in THF (1 mL) consumed 0.25 mL and 0.33 mL of *tert*-amylOMgCl·LiCl corresponding to 1.00 M.

2-Hydroxybenzaldehyde phenylhydrazone or benzoic acid and 4-(phenylazo)diphenylamine mixture can be used to confirm the absence of unreacted turbo Grignard.

1.2.9. Determination of the basicity concentration

1.2.9.1. C–Mg bases

An accurately weighed sample of 2-hydroxybenzaldehyde phenylhydrazone (40–100 mg) or benzoic acid (20–50 mg) and 4-(phenylazo)diphenylamine (indicator) are purged with vacuum/Ar and dissolved in THF (1 mL). The organomagnesium base is added slowly under manual mixing until yellow color solution turns orange (in the case of the hydrazone) or orange color solution turns deep red (in the case of the amine).

1.2.9.2. N–Mg bases

An accurately weighed sample of 2-hydroxybenzaldehyde phenylhydrazone (40–100 mg) or benzoic acid (20–50 mg) and thymolphthalein (indicator) are purged with vacuum/Ar and dissolved in THF (1 mL). The organomagnesium base is added slowly under manual mixing until yellow color solution turns orange (in the case of the hydrazone) or colorless solution turns deep blue (in the case of the thymolphthalein).

In the case of TMPMgCl·LiCl, only 2-hydroxybenzaldehyde phenylhydrazone reacts and turns orange.

1.2.9.3. O–Mg bases

An accurately weighed sample of a mixture of benzoic acid (20–50 mg) and thymolphthalein (indicator) are purged with vacuum/Ar and dissolved in 1 mL of THF. The organomagnesium base is added slowly under manual mixing until colorless solution turns blue.

1.2.10. NMR titration with 1,5-cyclooctadiene as a standard

A dried NMR tube is capped with rubber septum, purged with vacuum/Ar and tarred. 1,5-cyclooctadiene (COD, 30–50 mg) is loaded through the septum and the exact COD weight is determined. Toluene-d_8 (0.3 mL) and organomagnesium solution (0.6 mL) are added. The sample is mixed by manual agitation of the NMR tube.

$$[\mathrm{RMgX}]\ (\mathrm{M}) = \frac{[\mathrm{COD}]\ (\mathrm{mmol})}{\mathrm{Vol.\,RMgX}\ (\mathrm{mL})} \times \frac{\mathrm{RMgX\ Integration} \times \mathrm{COD\ H}}{\mathrm{COD\ Integration} \times \mathrm{RMgX\ H}}$$

1.3. ODR prototype procedures

Cartridges and bags assembly is identical for the generation of iPrMgCl·LiCl and HMDSMgCl·LiCl (Figure S5). The setup is composed by three bags and valvings thermally bonded together with 1/16” PFA tubing and custom made T-unions. Followed by a 95 mm long tubular reactor made of 3/8” PFA tubing, filled with magnesium chips/powder 1:1 (2.2 g), and a 110 mm long tubular reactor made of 3/8” PFA tubing, filled with LiCl (3.5 g), connected in-series. To avoid small solid particles to clog 1/16” tubing, pieces of fiber glass are added inside both extremes of the tubular reactors. Magnesium reactor is placed in one of the heater box. After the second column, a coil of 0.010” × 6 ft. (0.254 mm × 183 cm, ID × length) is added to generate back pressure and increase solvent boiling temperature. Finally, a custom made manifold allows to discard solvent used during system priming and undesired products formed during activation, collecting only during product generation. Flow rate is maintained at 0.5 mL/min adjusting pressure applied to the elastomer of the pumps.

The sequence of operation starts pumping solvent during 10 min by applying air pressure (14 psi) on the pump diaphragms. After 5 min, the heater starts to heat at 80 °C. Then, the activation solution is pumped for 10 min followed by the starting material solution. After 3 min pumping starting material solution, the air pressure applied on the pump diaphragms is gradually increased (95 psi) to maintain constant 0.5 mL/min flow rate (crude solution density increases during organomagnesium reagent formation). Once the color

indicator (2-hydroxybenzaldehyde phenylhydrazone) located in the waste bag turns orange, the manifold switches from waste to collection. Product is collected until the starting material bag is empty and the manifold switches back to waste. Finally, solvent is pumped during 20 min to wash the cartridge.

Activating solution: 1-bromo-2-chloroethane (345 mg, 0.2 mL, 2.4 mmol), chlorotrimethylsilane (TMSCl, 685 mg, 0.8 mL, 6.2 mmol) and DIBAL-H 1.0 M in toluene (4.5 mL, 4.5 mmol) in THF (4.5 mL).

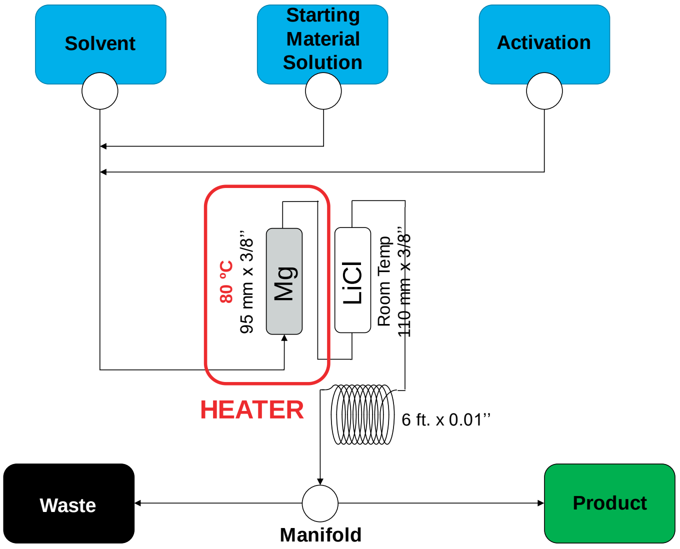

Figure S5. Scheme of the ODR prototype used for the generation of iPrMgCl·LiCl and HMDSMgCl·LiCl.

1.3.1. Turbo Grignard (iPrMgCl·LiCl)

Solvent bag: THF (20 mL).
Starting material solution: 2-chloropropane (4.2 mL, 45 mmol) in THF (16.3 mL).
Product: Isopropylmagnesium chloride lithium chloride complex 1.9 M (15–17 mL).

1.3.2. Amidomagnesium chloride lithium chloride complex derived from HMDS (HMDSMgCl·LiCl)

Solvent bag: THF (10 mL) and toluene (10 mL).
Starting material solution: 2-chloropropane (3.9 mL, 42 mmol), HMDS (8.8 mL, 42 mmol) in THF (11.1 mL) and toluene (11.1 mL).
Product: HMDS amidomagnesium chloride lithium chloride complexe 1.0 M (30–32 mL)

2. Results and discussion

2.1. Grignard reagents via magnesium packed beds

We used 2-bromo and 2-chloropropane to screen and optimize conditions. Directly after activation, iPrX solution in THF was pumped at 0.5 mL/min flow rate through the column (≈4 min residence time, t_R). Conversion of iPrBr into the corresponding organomagnesium reagent was performed at 25 °C. Heat generated during the reaction was not fully dissipated by the manifold. Higher temperatures were observed when more concentrated organohalide solutions were used. Once in a steady state, the outcome solution was collected in a flask under argon.

For the iPrBr initial concentration ≥ 1.0 M, an oversaturated organomagnesium solution was obtained, leading to crystallization in the collection flask (Table S4, entries 1 and 2). The organohalide initial concentration was reduced to 0.9 M to avoid crystallization, yielding to iPrMgBr 0.75 M (82%) as a pale orange clear solution (Table S4, entry 3). The same conditions were used with iPrCl but no Grignard reagent was obtained (Table S4, entry 4). The temperature was increased and the best result was obtained at 80 °C, yielding iPrMgCl 0.78 M (87%, Table S4, entry 7). Since iPrMgCl (dimer in ether at all concentration)[7] is more soluble than the corresponding bromide (dimers, trimers, and higher polymers in ether at higher concentration: 0.5–1 M),[7] we were able to increase the initial concentration up to 2.5 M, yielding to iPrMgCl 2.23 M (89%) as pale orange clear solution (Table S4, entry 8).

Table S4. Reaction of 2-bromo and 2-chloropropane with activated magnesium. Temperature optimization.[a]

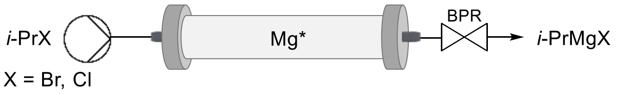

Entry	RX	T (C)	BPR (psi)	[RX] (M)[b]	[RMgX] (M)[c]	Yield (%)
1	*i*-PrBr	25	20	1.2	0.95	79[d]
2	*i*-PrBr	25	20	1.0	0.81	81[d]
3	*i*-PrBr	25	20	0.9	0.75	82
4	*i*-PrCl	25	20	0.9	0	0
5	*i*-PrCl	40	20	0.9	0.20	22
6	*i*-PrCl	60	100	0.9	0.72	80
7	*i*-PrCl	80	100	0.9	0.78	87
8	*i*-PrCl	80	100	2.5	2.23	89

[a] iPrX solution in THF (15 mL) was pumped at 0.5 mL/min flow rate through a packed bed column (ID = 10 mm) of activated Mg* (4 g, 161 mmol, powder 20–230 mesh). [b] Quantitative conversion. [c] Determined by colorimetric titration (duplicate) of overall iPrMgX solution (≈10 mL) collected in a steady state under argon. [d] iPrMgBr solution crystallized in collection flask.

During iPrMgCl optimization, we observed the formation of a black residue at the base of the column. For example, when ≈30 mL of iPrCl 2.5 M (≈75 mmol) solution was pumped through the column (Mg powder, ≈5 g, ≈200 mmol, 98%, 20–230 mesh), 3 cm of the 5 cm of Mg* were converted into the black solid and increase of the system clogged after 75% of iPrCl solution volume was flowed, limiting the amount of organomagnesium generated due to pressure drop increase. Analysis via X-ray Photoelectron Spectroscopy (XPS, Figure S6) of the black residue, after been washed with pentane at 0 °C and dried under vacuum, revealed the presence of magnesium, oxygen, carbon and chlorine.[8-9]

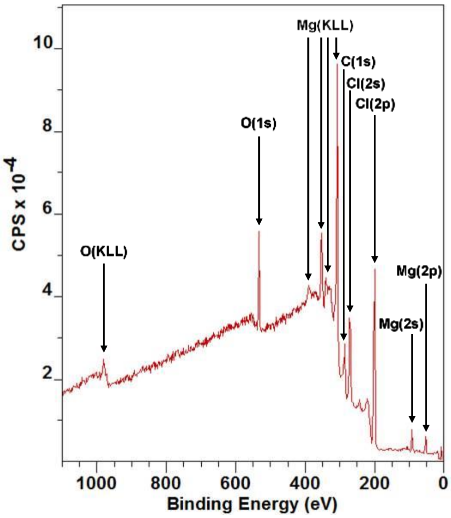

Figure S6. XPS analysis of the black residue.

Black residue formation was investigated using different Mg chips and powder ratios: 0:1, 1:3, 1:1, 3:1, and 1:0 (Figure S7). We found that 2 equivalents of Mg chips/powder 1:1 molar ratio were optimal to convert 1 equivalent of iPrCl (2.5 M, 40 mL, 100 mmol) into iPrMgCl (2.43 M, 97%) with no clogging. The Mg chips/powder mixture significantly increased the yield obtained, from 89% with exclusively Mg powder (Table S4, entry 8) to 97% with Mg chips/powder 1:1 wt % mixture.

After having flowed 40 mL of iPrCl 2.5 M (100 mmol, 1 equiv) through Mg* (200 mmol, 2 equiv), iPrMgCl concentration started to drop independently the chips/powder ratio used (Figure S7A). Powder ratios higher than 50% yielded the same clogging outcome as described above with only Mg* powder. Inversely, at chip ratios higher than 50% unreacted iPrCl was recovered leading to lower yields (Figure S7A). Back pressure evolution was measured during these experiments and we observed that pressure increases proportionally

with the amount of Mg powder present in the column (Figure S7B). Despite pressure increase at the end of the experiment, the 1:1 chip/powder system provided much more consistent results over relatively large volumes of organohalide converted.

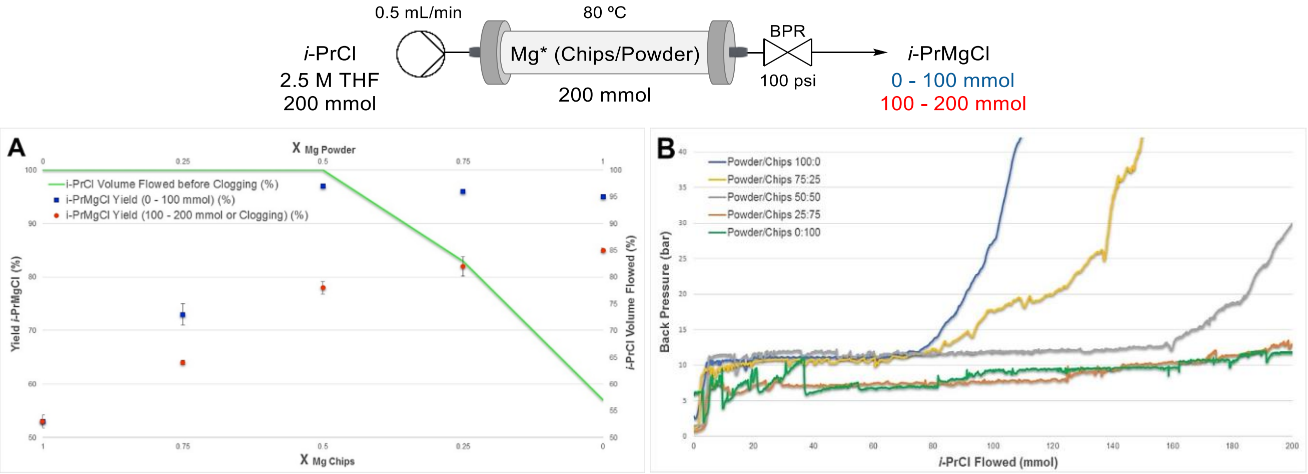

Figure S7. Formation of iPrMgCl using different magnesium chips/powder ratios: 0:1, 1:3, 1:1, 3:1, and 1:0. (A) Yield of iPrMgCl (blue/red dots) and volume of iPrCl solution consumed before clogging (green line). (B) Back-pressure evolution measured by a Vapourtec flow system.

Yield drop after having flowed 20 mL of iPrCl 2.5 M (100 mmol, 1 equiv) through Mg* (200 mmol, 2 equiv) was attributed to channeling through the packed bed, thus reducing the surface area of reactive Mg* in contact with iPrCl. We decided to study this channeling effect comparing a well packed with an unpacked *free-flowing* Mg column (chips/powder 1:1 ratio). Packed columns were prepared as described in Section 1.*2*.1, and packing was done manually screwing the plunger until it stopped moving down. Unpacked columns were identical but plunger was stopped 2 mm before Mg bed. Eight fractions of iPrMgCl solution (0–200 mmol) were collected and the concentrations was determined by colorimetric titration. During the reactions, Mg columns consumption were recorded using a 4K webcam and snapshots of these videos were added to the graphs (Figure S8). We can see that the well packed Mg column did not consumed all the Mg (grey solid left) due to channeling (Figure S8A). On the contrary, unpacked Mg, after consumption of a certain amount of metal, behaved more as fluidized bed, allowing iPrCl to be in contact with a larger Mg* surface (Figure S8B). *Free-flowing* Mg column gave the best yields: 98% and 70% respectively for the first (0–100 mmol) and second (100–200 mmol) equivalent of iPrCl flowed through Mg*. Mixing of Mg particles avoid channeling leading to a more progressive yield drop of the second equivalent (100–200 mmol). This yield evolution study confirmed that 2 equivalents of Mg* are indeed needed since iPrMgCl yield of the second equivalent continuously decreased even with unpacked Mg column. Chips accumulation at the base prevents clogging. We noticed that the central fractions were slightly over-concentrated (> 100%). The green box on Figure S8B approximately indicates steady state window. The concentration variation in this window is (98±5)% and it is most probably due to measurement errors produced during concentration colorimetric determination.

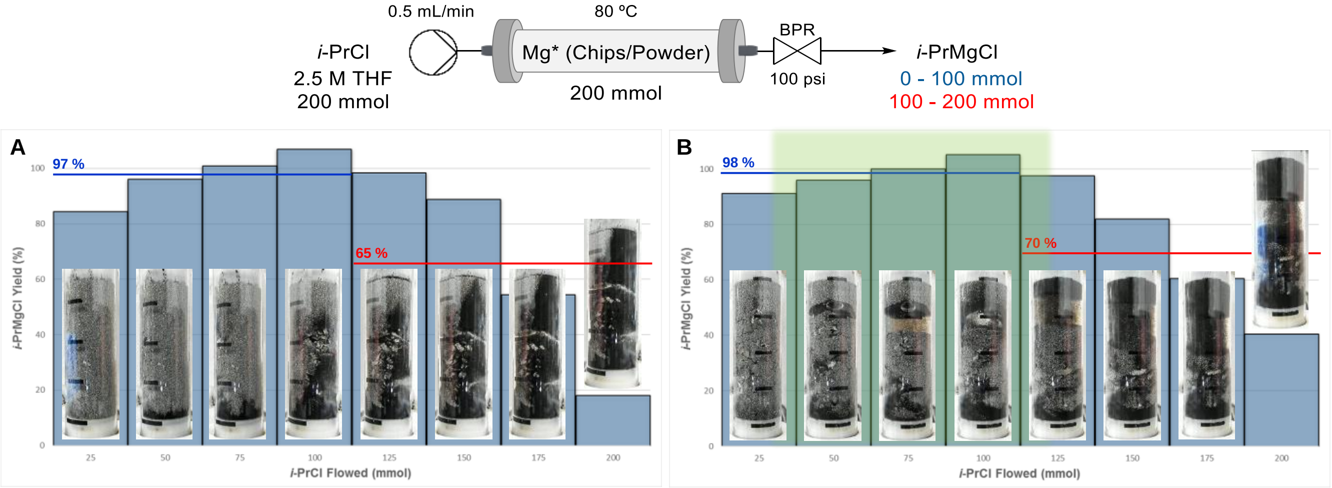

Figure S8. Yield evolution of iPrMgCl using: (A) a well-packed magnesium column (chips/powder 1:1 Ratio); (B) an unpacked free-flowing magnesium column (chips/powder 1:1 ratio, the green box approximately indicates the steady state window). The snapshot of the magnesium consumption of each fraction reveals the different behaviors between a packed bed (A) and a fluidized bed (B).

During Grignard scope exploration, we investigated the formation benzylmagnesium chloride. We expected a low amount of Wurtz-type coupling by-product since continuous flow synthesis should reduce the presence of unreacted chloromethylbenzene with benzyl Grignard. Unfortunately, using our standard conditions (THF, 80 °C, 0.5 mL/min) only 0.41 M (34%) of BnMgCl was obtained (Table S5, entry 1). In order to slow down the reaction, temperature was reduced to 25 °C producing a yield increase up to ≈50% (Table S5, entries 2 and 4). The faster flow rate did not improve further the yield (Table S5, entries 5 and 6). Zhang et al. demonstrated that Grignard reaction of benzyl chloride in 2-MeTHF reduced the formation Wurtz coupling by-product.[10] The reaction was repeated in 2-

MeTHF at 25 °C and 80% of BnMgCl was formed but with unreacted BnCl present in the mixture (Table S5, entry 7). Reactor was heated to 60 °C and full conversion was achieved but the mixture became less soluble. A mixture of 2-MeTHF/THF (9:1) was found to be optimal to reduce the formation of 1,2-diphenylethane, yielding BnMgCl 0.99 M (83%, Table S5, entry 10).

Table S5. Reaction of chloromethylbenzene with activated magnesium. Optimization under flow conditions to reduce the Wurtz-type byproduct.[a]

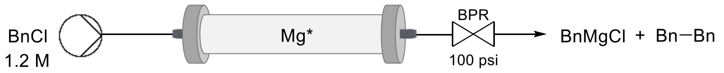

Entry	Solvent	T (C)	ID (mm)	Flow rate (mL/min)	[BnMgCl] (M)	Yield (%)[b]
1	THF	80	10	0.5	0.41	34
2	THF	25	10	0.5	0.67	56
3	THF[c]	25	10	0.5	0.22	18
4	THF	25	15	0.5	0.66	55
5	THF	25	10	2.0	0.62	52
6	THF	25	15	5.0	0.20	17[d]
7	2-MeTHF	25	10	0.5	0.96[e]	80[d]
8[f]	2-MeTHF	60	10	0.5	0.94[e]	74
9	2-MeTHF/THF (3:1)	60	10	0.5	0.85	71
10	2-MeTHF/THF (9:1)	60	10	0.5	0.99	83

[a] BnCl solution (15 mL) was flowed through a column of Mg* (2 equiv) chips/powder 1:1 ratio. [b] Determined by colorimetric titration (duplicate) of overall BnMgCl solution (≈10 mL) collected in a steady state under argon. [c] LiCl 0.5 M solution in THF. [d] RX conversion < 100%. [e] Not totally soluble. [f] Mg and LiCl bicomponent column used. ID = internal diameter.

2.2. Turbo Grignards via stratified packed-bed columns containing magnesium and LiCl

Clogging is a common concern in flow chemistry and during our scope exploration the organomagnesium reagents concentration obtained was mostly limited by their solubility. Knochel has demonstrated countless times the exceptional ability of lithium chloride to solubilize organometallic reagents and to increase reactivity most probably due to disaggregation of oligomers.[11-12] We used this approach to overcome the solubility issue under continuous conditions. First, we verified that similar results are achieved in presence and absence of LiCl for EtMgBr formation. Since organomagnesium halide lithium chloride complexes are believed to be RMgX·LiCl 1:1 dimer, bromoethane was dissolved in LiCl 0.5 M THF solution in 1:1 molar ratio.[13] The results demonstrated similar EtMgBr yields in presence or absence of LiCl, 82% and 80% respectively (Table S6, entries 1 and 2). Unfortunately, LiCl solubility in THF is ≈0.5 M. So, at RX concentrations higher than 0.5 M less than 1 equivalent of LiCl can be added. However, similar yields were obtained for 1 M and 1.5 M EtBr solution even if RX:LiCl ratio was 2:1 and 3:1 respectively (Table S6, entries 3–6). In the case of 1.5 M bromoethane solution in absence of LiCl, an insoluble mixture was obtained (Table S6, entry 5). Addition of LiCl 0.5 M THF solution allowed the collection of 1.27 M (85%) EtMgBr·LiCl stable solution (Table S6, entry 6).

Table S6. Reaction of 1-bromoethane with a column of activated magnesium at 0.5, 1.0, and 1.5 M in presence and absence of LiCl in solution.[a]

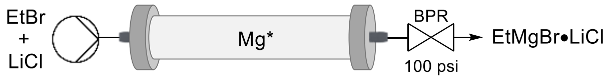

Entry	[RX] (M)[b]	LiCl (eq.)	RX:LiCl	[EtMgBr•LiCl] (M)	Yield (%)[c]
1	0.5	0	-	0.40	80
2	0.5	1.0	1:1	0.41	82
3	1.0	0	-	0.78	78
4	1.0	0.5	2:1	0.81	84
5	1.5	0	-	1.34	89[d]
6	1.5	0.33	3:1	1.27	85

[a] EtBr was dissolved in THF or in LiCl 0.5 M THF solution and pumped at 0.5 mL/min flow rate and 25 °C through an activated Mg* (2 equiv) column (ID = 10 mm) chips/powder ratio 1:1. [b] Quantitative RX conversion. [c] Determined by titration of overall collected solution in a steady state;
[d] iPrMgBr solution crystallized in collection flask.

3. References

[1] https://www.vapourtec.com/products/r-series-flow-chemistry-system-overview/
[2] https://www.dibaind.com/labware/#omnifitsupsup-labware-glasschromatography-columns
[3] L. Huck, A. de la Hoz, A. Díaz-Ortiz, J. Alcázar, *Org. Lett.* **2017**, *19*, 3747-3750.
[4] B. E. Love, E. G. Jones, *J. Org. Chem.* **1999**, *64*, 3755-3756.
[5] S. Blumberg, S. F. Martin, *Tetrahedron Lett.* **2015**, *56*, 3674-3678.
[6] T. R. Hoye, B. M. Eklov, M. Voloshin, *Org. Lett.* **2004**, *6*, 2567-2570.
[7] E. C. Ashby, M. B. Smith, *J. Am. Chem. Soc.***1964**, *86*, 4363-4370.
[8] M. Kapilashrami, J. Xu, K. V. Rao, L. Belova, E. Carlegrim, M. Fahlman. *J. Phys.: Condens. Matter.* **2010**, *22*, 345004-345008.
[9] https://xpssimplified.com/periodictable.php.
[10] A. Kadam, M. Nguyen, M. Kopach, P. Richardson, F. Gallou, Z.-K. Wane, W. Zhang, *Green Chem.* **2013**, *15*, 1880-1888.
[11] T. Klatt, J. T. Markiewicz, C. Sämann, P. Knochel, *J. Org. Chem.* **2014**, *79*, 4253-4269.
[12] D. S. Ziegler, B. Wei, P. Knochel, *Chem. Eur. J.* **2019**, *25*, 2695-2703.
[13] A. Krasovskiy, P. Knochel, *Synthesis* **2006**, *5*, 890-891.

4. Author contributions

D.T.M. and A.A. conceived the idea. A.A. and K.S. designed and built the ODR system and cartridges. K.S. programmed the software. M.B. developed and implemented the stratified bicomponent packed bed reactors. M.B. optimized and performed organomagnesium reactions. M.B. contributed to data acquisition and analysis. M.B., D.T.M. and A.A. wrote the manuscript.

5. NMR Spectra

5.1. Titration of turbo Grignard (iPrMgCl•LiCl) in THF with COD in toluene-d_8 (600 MHz)

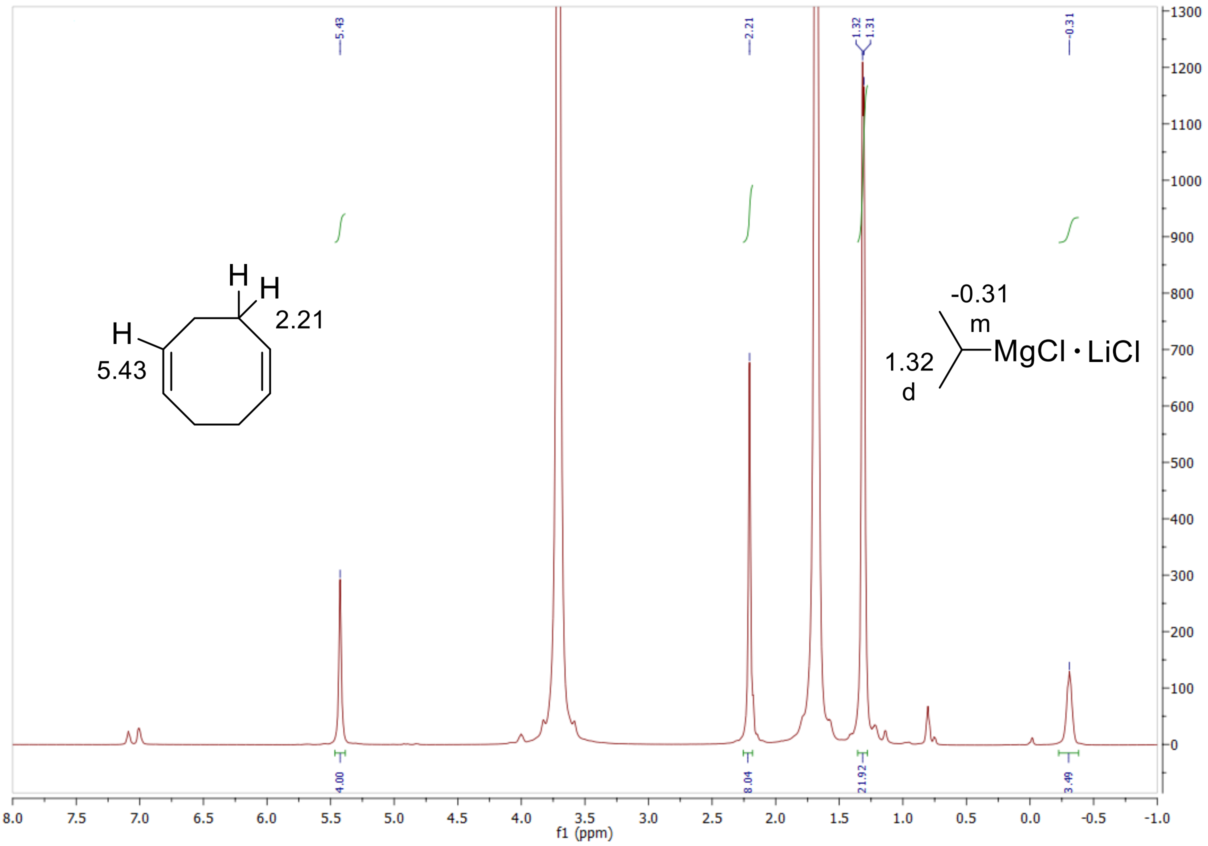

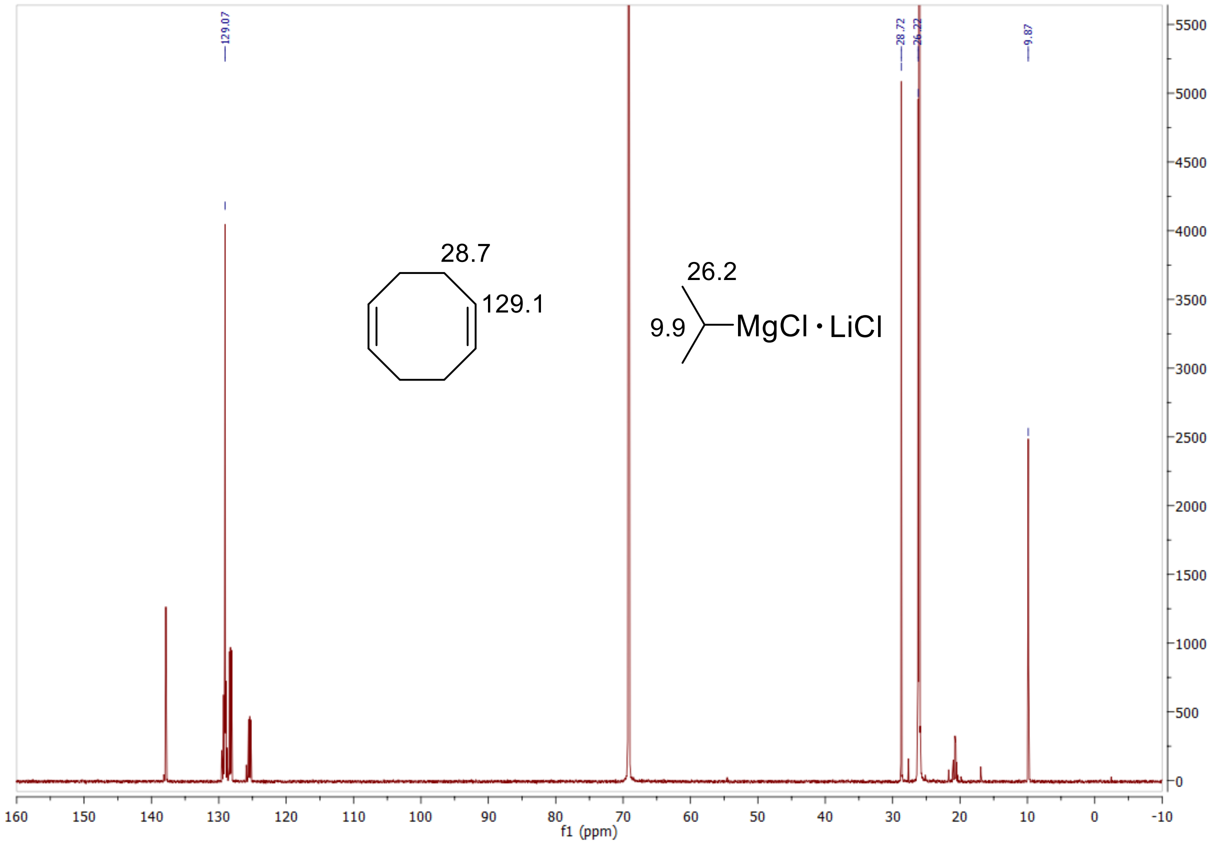

5.2. Titration of HMDS amidomagnesium chloride LiCl complex (HMDSMgCl•LiCl) in THF/Tol. with COD in toluene-d_8 (600 MHz)

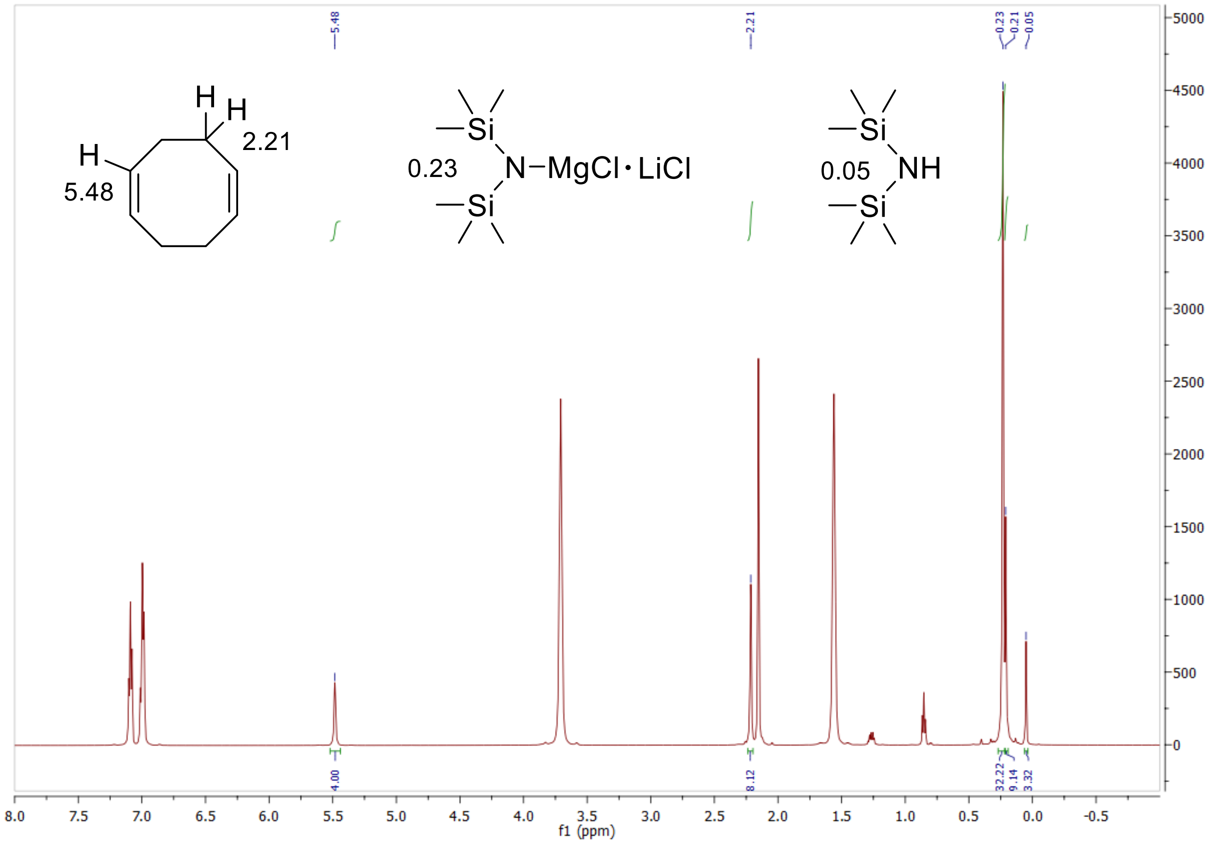

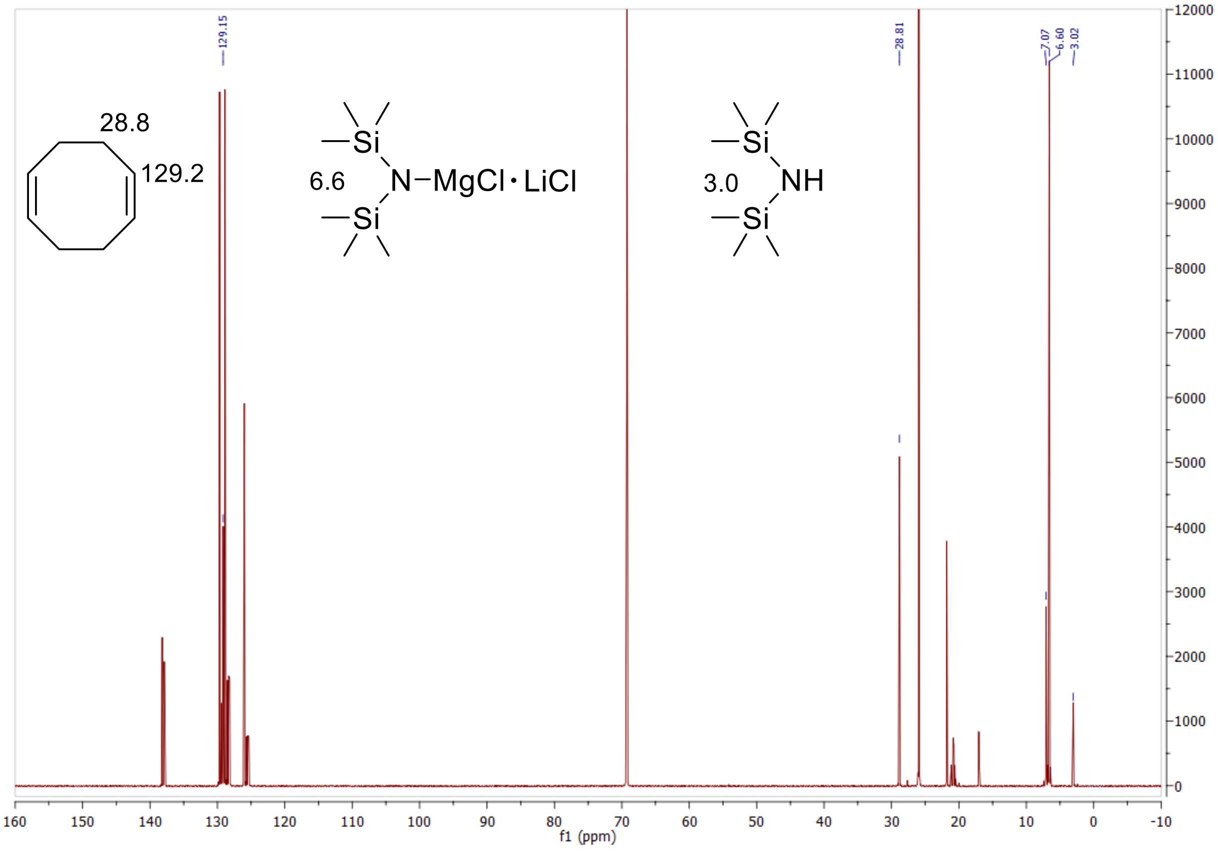

5.3. Turbo Grignard (*i*-PrMgCl•LiCl) generated with ODR prototype in THF with COD in toluene-d_8 (300 MHz)

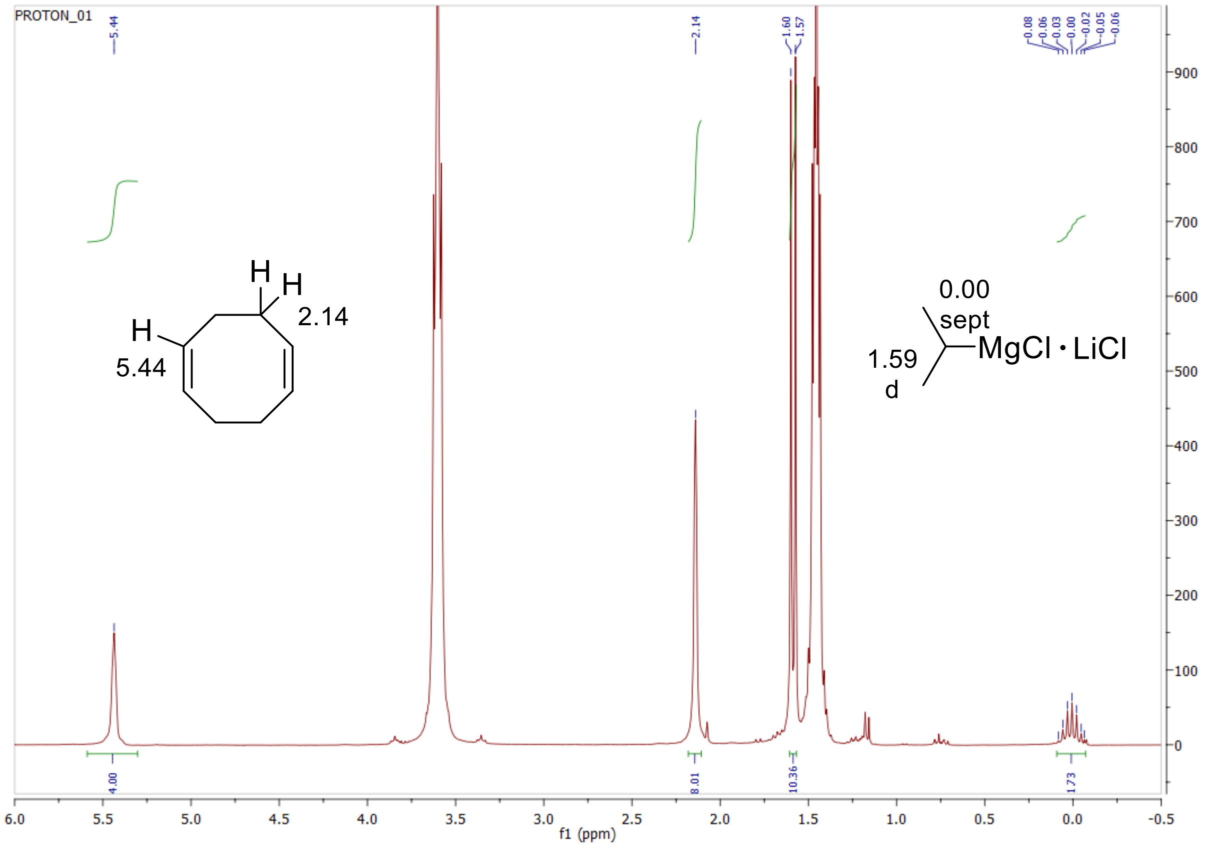

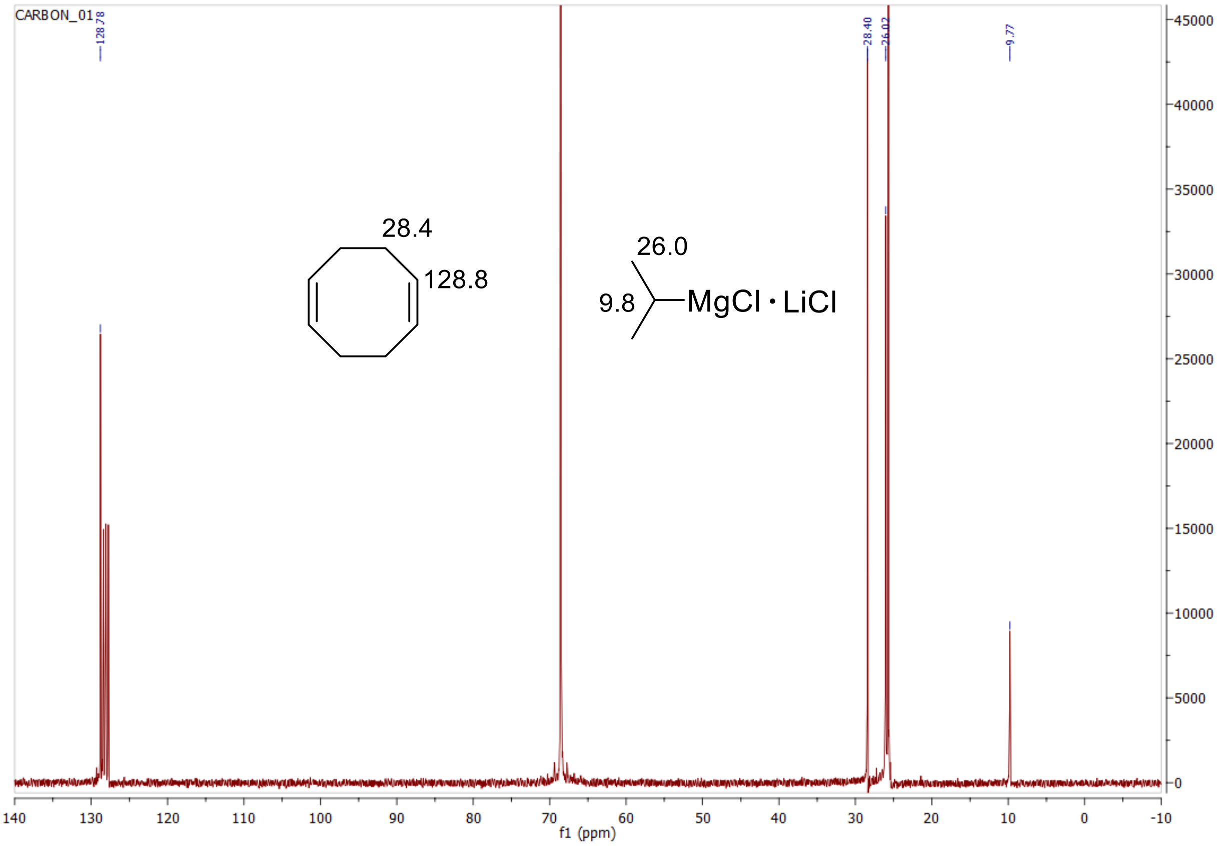

5.4. HMDS Amidomagnesium chloride LiCl complex (HMDSMgCl•LiCl) generated with ODR prototype in THF/Tol. with COD in toluene-d_8 (300 MHz)

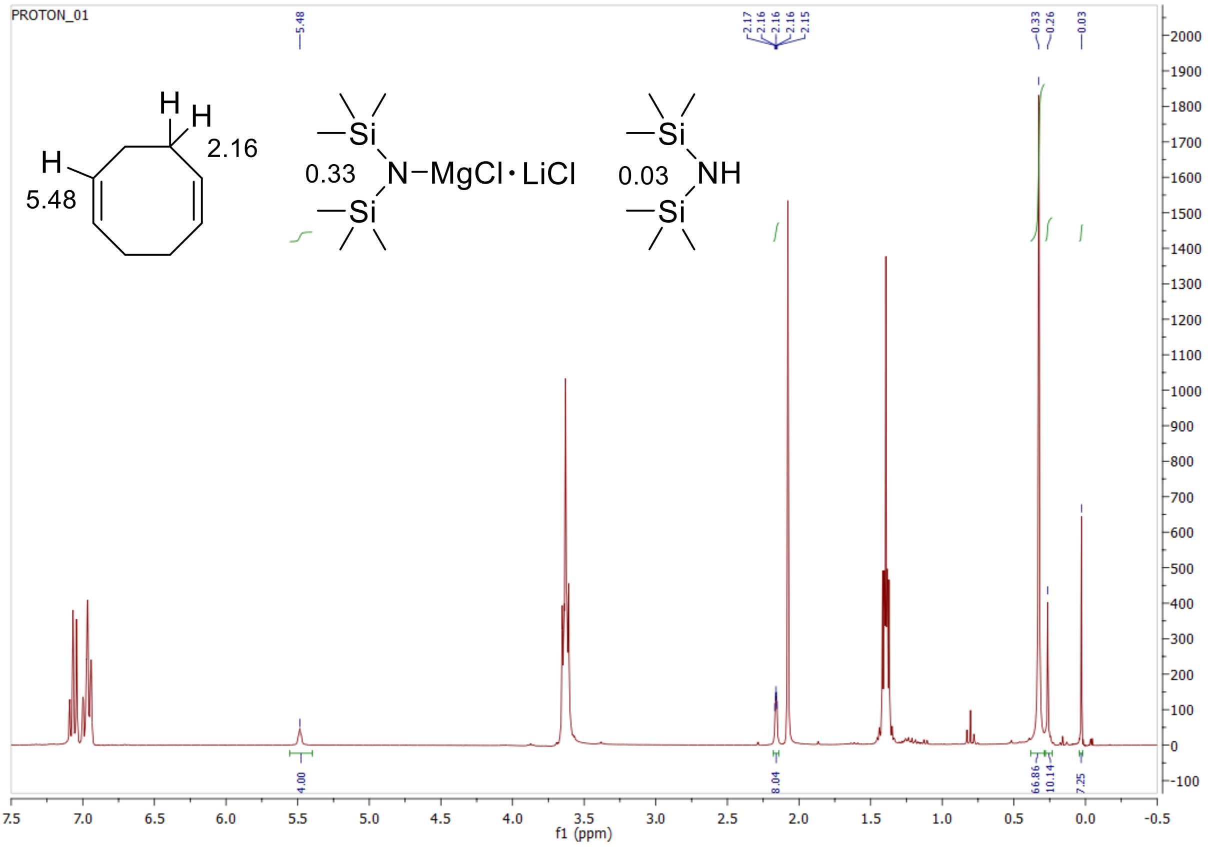

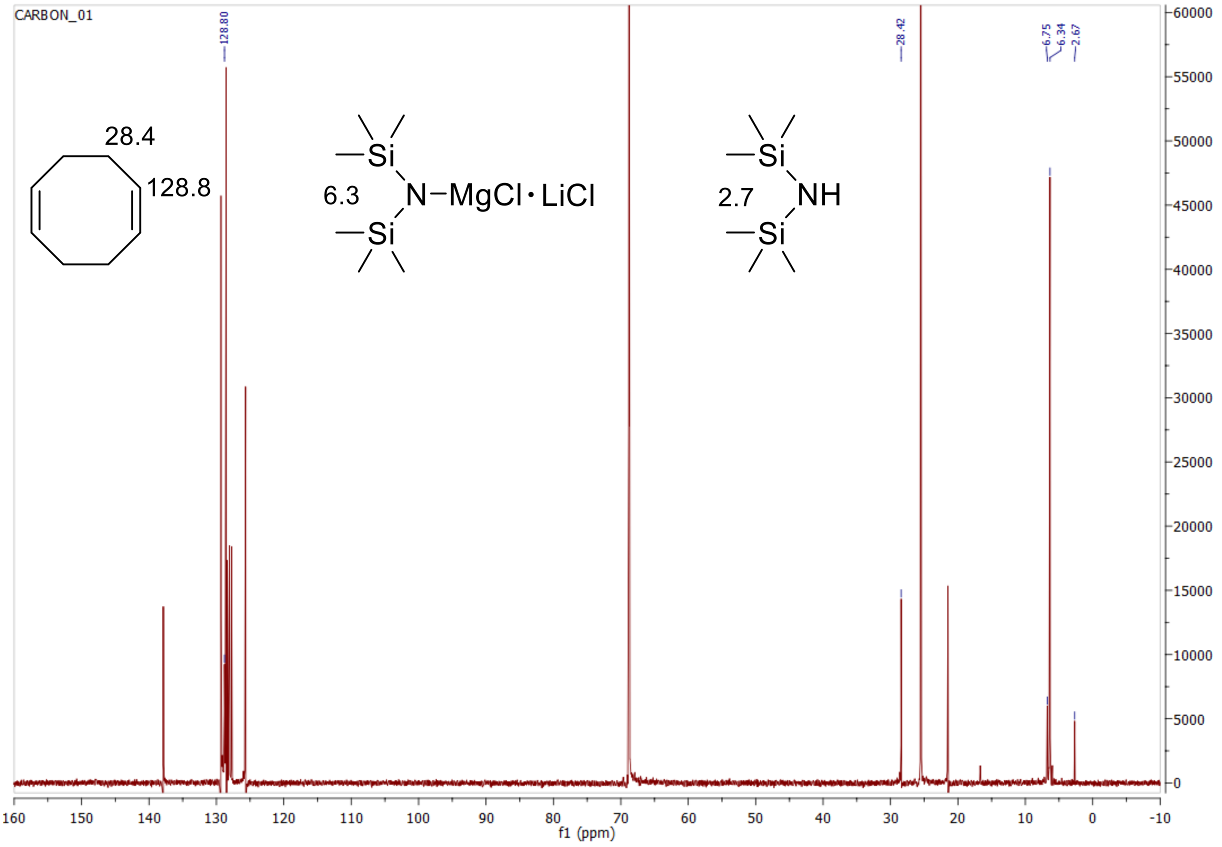

Supplement: File 1 — Additional experimental data. [file Beilstein_J_Org_Chem-16-1343-s001.pdf]
